# Blastocyst transfer in mice alters the placental transcriptome and growth

**DOI:** 10.1530/REP-19-0293

**Published:** 2019-11-18

**Authors:** Katerina Menelaou, Malwina Prater, Simon J Tunster, Georgina E T Blake, Colleen Geary Joo, James C Cross, Russell S Hamilton, Erica D Watson

**Affiliations:** 1 Department of Physiology, Development, and Neuroscience, University of Cambridge, Cambridge, UK; 2 Centre for Trophoblast Research, University of Cambridge, Cambridge, UK; 3 Transgenic Services, Clara Christie Centre for Mouse Genomics, University of Calgary, Calgary, Alberta, Canada; 4 Department of Comparative Biology and Experimental Medicine, University of Calgary, Calgary, Alberta, Canada; 5 Department of Genetics, University of Cambridge, Cambridge, UK

## Abstract

Assisted reproduction technologies (ARTs) are becoming increasingly common. Therefore, how these procedures influence gene regulation and foeto-placental development are important to explore. Here, we assess the effects of blastocyst transfer on mouse placental growth and transcriptome. C57Bl/6 blastocysts were transferred into uteri of B6D2F1 pseudopregnant females and dissected at embryonic day 10.5 for analysis. Compared to non-transferred controls, placentas from transferred conceptuses weighed less even though the embryos were larger on average. This suggested a compensatory increase in placental efficiency. RNA sequencing of whole male placentas revealed 543 differentially expressed genes (DEGs) after blastocyst transfer: 188 and 355 genes were downregulated and upregulated, respectively. DEGs were independently validated in male and female placentas. Bioinformatic analyses revealed that DEGs represented expression in all major placental cell types and included genes that are critical for placenta development and/or function. Furthermore, the direction of transcriptional change in response to blastocyst transfer implied an adaptive response to improve placental function to maintain foetal growth. Our analysis revealed that CpG methylation at regulatory regions of two DEGs was unchanged in female transferred placentas and that DEGs had fewer gene-associated CpG islands (within ~20 kb region) compared to the larger genome. These data suggested that altered methylation at proximal promoter regions might not lead to transcriptional disruption in transferred placentas. Genomic clustering of some DEGs warrants further investigation of long-range, cis-acting epigenetic mechanisms including histone modifications together with DNA methylation. We conclude that embryo transfer, a protocol required for ART, significantly impacts the placental transcriptome and growth.

## Introduction

More than seven million babies worldwide have been born using some form of assisted reproduction technology (ART) largely as a treatment approach to infertility (European Society of Human Reproduction and Embryology (ESHRE) 2018 February 18. *ART Fact Sheet*. Retrieved from https://www.eshre.eu/. Grimbergen, Belgium). ART includes a range of procedures with varying degrees of invasiveness (e.g., superovulation, *in vitro* fertilisation (IVF), intracytoplasmic sperm injection, embryo culture, embryo biopsy, gamete and embryo vitrification, and blastocyst transfer). While ART is generally safe, growing evidence suggests that individuals born using these technologies are at an increased risk of intrauterine growth restriction, perinatal complications (Quinn & Fujimoto 2016), and/or developing cardiovascular disease later in life (Tararbit *et al.* 2013, Valenzuela-Alcaraz *et al.* 2013, Liu *et al.* 2015, Guo *et al.* 2017). Since optimal placental function is required for normal foetal growth and development, it is predicted that placenta pathologies are responsible for some of the adverse pregnancy outcomes associated with ART (Delle Piane *et al.* 2010, Thomopoulos *et al.* 2013, Choux *et al.* 2015). Indeed, ART pregnancies were overrepresented in the highest quartile of placental weight and underrepresented in the highest quartile of birthweight (Haavaldsen *et al.* 2012).

To explore the effects of ART on placental structure and function, animal models have been utilised. Similar to humans, the mouse placentation site is composed of three major layers: the outer maternal layer, which includes decidual cells of the uterus, maternal immune cells, and the maternal vasculature that brings blood to and from the implantation site; the metabolic ‘junctional’ region, containing many endocrine cells and which attaches the placenta to the uterus through the invasion of trophoblast cells; and an inner layer composed of highly branched villi required for nutrient, gas, and waste exchange between maternal and foetal circulations (Watson & Cross 2005). Defects in placenta development and/or function have repercussions for foetal growth and health (Watson & Cross 2005, Perez-Garcia *et al.* 2018). IVF and/or superovulation in mice are associated with large placentas with reduced vascular density and altered nutrient transport at late gestation to produce normal-sized or growth-restricted fetuses (Delle Piane *et al.* 2010, Bloise *et al.* 2012, Weinerman *et al.* 2017). Indeed, sub-optimally formed placentas might undergo counter-balancing mechanisms leading to adaptive responses (Choux *et al.* 2015). How and when this dialogue occurs is unclear.

The mechanism through which ART influences the formation and function of the maternal-fetal interface is not well understood. The vast majority of studies have focused on CpG methylation of imprinted genomic loci (Khosla *et al.* 2001, Mann *et al.* 2004, Fortier *et al.* 2008, Rivera *et al.* 2008, Fauque *et al.* 2010, Wang *et al.* 2010, Bloise *et al.* 2012, de Waal *et al.* 2014). Genomic imprinting is an epigenetic phenomenon in mammals whereby a small number of genes are expressed in a parent-of-origin-specific manner, a process that is regulated by DNA methylation. Many imprinted genes are expressed in the placenta and are important for its development and function (Tunster *et al.* 2013). Directed analysis of imprinted regions can act as a convenient read-out of functional DNA methylation changes across the genome (Padmanabhan *et al.* 2013). Therefore, studies showing altered DNA methylation at imprinted loci after ART hypothesise that these technologies might influence the establishment of DNA methylation genome-wide with consequences for placental cell differentiation (Choux *et al.* 2015). Dysregulation of other epigenetic mechanisms (e.g., histone modifications, non-coding RNA expression) are largely unstudied in the context of ART.

One procedure that all ARTs have in common is the transfer of the blastocyst into the uterus of a recipient female. While many ART studies in mice have taken into account the potential effects of blastocyst transfer (Kholsa *et al.* 2001, Delle Piane *et al.* 2010, Fauque *et al.* 2010, Bloise *et al.* 2012), the impact of this procedure alone on placentation site growth and transcription is not well understood. Here, we show that blastocyst transfer in mice has a stark impact on transcriptional regulation and likely influences placental efficiency even as the placenta matures. Furthermore, our genome-wide approach enabled us to determine that changes in DNA methylation at proximal promoter regions may not cause transcriptional disruption. Therefore, a long-range study of cis-acting epigenetic mechanisms in addition to DNA methylation is required.

## Materials and methods

### Mice

C57Bl/6 conceptuses were generated by natural mating of C57Bl/6 mice at 7–10 weeks of age. No hormones were used. (C57Bl/6 × DBA/2) F1 hybrid (B6D2F1) female mice were mated at 7–10 weeks of age with vasectomized C57Bl/6 males to generate a pseudopregnant state. B6D2F1 mice have 50% genetic similarity to C57Bl/6 mice and were used because C57Bl/6 females are notoriously poor recipients for blastocyst transfer. A broad range of genetic backgrounds has been used for donor and recipient mice across ART studies (Mann *et al.* 2004, Fortier *et al.* 2008, Piane *et al.* 2010, Wang *et al.* 2010, Chen *et al.* 2015), including hybrid recipient females as in our study (Kholsa *et al.* 2001, Fauque *et al.* 2010, Bloise *et al.* 2012). C57Bl/6 conceptuses that were derived by natural mating and did not undergo the transfer process were used as controls. Noon of the day that the copulatory plug was detected was considered embryonic (E) day 0.5. Mice were killed via cervical dislocation. All experiments were performed in accordance with the Canadian Council on Animal Care and the University of Calgary Committee on Animal Care (protocol number M06109). This research was also regulated under the Animal (Scientific Procedures) Act 1986 Amendment Regulations 2012 following ethical review by the University of Cambridge Animal Welfare and Ethical Review Body.

### Blastocyst transfers

Conceptuses from the blastocyst transfer experiment were generated as previously described (Padmanabhan *et al.* 2013). Briefly, using M2 media (Sigma-Aldrich), embryos were flushed at E3.25 from the oviducts and uteri of C57Bl/6 females. Embryos were cultured in KSOM media (Millipore) microdrops covered in mineral oil (Millipore) at 37°C for no more than 30 min. This unavoidable culture period allowed for the surgical preparation of the recipient female. Embryos were transferred by injection into the oviducts of pseudopregnant B6D2F1 recipients 2.5 days after mating them with vasectomized C57Bl/6 males. Litters were never pooled.

### Dissections and phenotyping

All conceptuses were dissected at E10.5. For transferred litters, the timing of dissection corresponded to the staging of the recipient female (Ueda *et al.* 2003). Implantation sites were dissected away from the uterine myometrium in cold 1× phosphate buffered saline. Embryos and placentas were rigorously scored for gross phenotypes (see below and Padmanabhan *et al.* 2013), photographed, weighed, and snap frozen in liquid nitrogen for storage at −80°C. Similar to other ART studies on mouse placenta (Fortier *et al.* 2008, Rivera *et al.* 2008, Fauque *et al.* 2010, Wang *et al.* 2010, Bloise *et al.* 2012), whole placentas including the mesometrial decidua were assessed. Retaining the decidua is important for sample consistency since reliably removing the decidua without influencing parietal trophoblast giant cell (TGC) numbers is difficult. It also contains invading trophoblast cells (Simmons *et al.* 2008) that are important to assess.

For phenotype scoring, defective embryo growth was determined by assessing crown-rump length and somite pairs. Crown-rump lengths that were greater or less than two standard deviations from the mean crown-rump length of C57Bl/6 controls were distinguished as growth enhancement or restriction, respectively. Somite staging was determined according to e-Mouse Atlas Project (http://www.emouseatlas.org): 30–39 somite pairs were considered the normal range for E10.5 and somite pairs <30 indicated developmental delay. Embryos were also assessed for the appearance of haemorrhage, resorptions, twinning, and congenital malformations including defective neural tube closure, abnormal heart loop directionality, and/or presence of pericardial oedema or heart enlargement. Placentas were grossly scrutinized for the presence and orientation of chorioallantoic attachment and haemorrhage. Sex of conceptuses was determined by polymerase chain reaction (PCR) genotyping of yolk sac DNA using reported methods (Chuma & Nakatsuji 2001, Tunster 2017). Images were obtained using a Zeiss SteREO Discovery V8 microscope with an AxioCam MRc5 camera and AxioVision 4.7.2 software (Carl Zeiss Ltd).

### RNA and DNA extraction

Whole placentas were homogenized using lysing matrix D beads (MP Biomedicals, Carlsbad, USA). For quantitative reverse transcription-PCR (qRT-PCR) analysis, total RNA was extracted using TRIzol reagent (Sigma-Aldrich) according to the manufacturer’s instructions. For methylation analysis, DNA and RNA were extracted using the AllPrep DNA/RNA kit (QIAGEN). All RNA extracts were treated with DNAse I (Thermo Scientific).

### Transcriptome analysis

RNA libraries were prepared from whole placentas of non-transferred and transferred C57Bl/6 conceptuses at E10.5. Four male placentas taken from 3 to 4 litters were assessed per experimental group. Placentas chosen for analysis were associated with phenotypically normal embryos (i.e., the embryos displayed normal crown-rump lengths and somite pair counts and were absent of congenital malformations). Library preparation and sequencing was performed by Cambridge Genomic Services, Department of Pathology, University of Cambridge. The concentration and purity of RNA was determined by a SpectroStar spectrophotometer (BMG LABTECH, Aylesbury, UK) and an Agilent Tapestation Bioanalyzer (Aligent Technologies LDA UK Ltd) determined RNA integrity. Libraries were prepared using 200 ng of total RNA and TruSeq stranded mRNA Library Preparation kit (Illumina, Chesterford, UK). A unique index sequence was added to each RNA library to allow for multiplex sequencing. Libraries were pooled and sequenced on the Illumina NextSeq500 platform with 75-base-pair single-end reads. Sequencing was performed in duplicate to provide >18 million reads per sample. To monitor sequencing quality control, 1% PhiX Control (Illumina) spike-in was used. Quality control of Fastq files was performed using FastQC and fastq_screen. Sequences were trimmed with Trim Galore! and aligned to GRCm38 mouse genome using STAR aligner. Alignments were processed using custom ClusterFlow (v0.5dev) pipelines and assessed using MultiQC (0.9.dev0). Gene quantification was determined with HTSeq-Counts (v0.6.1p1). Additional quality control was performed with rRNA and mtRNA counts script, feature counts (v 1.5.0-p2) and qualimap (v2.2). Differential gene expression was performed with DESeq2 package (v1.22.2, R v3.5.2). Read counts were normalised on the estimated size factors. Principle component analysis was performed on the rlog transformed count data for the top 5000 most variable genes. Heatmaps were generated with ‘ComplexHeatmap’ R package (v 1.20.0). Karyoplots were generated with karyoploteR (v1.8.8).

### Data availability

The RNA-sequencing data are accessible through ArrayExpress EMBL-EBI accession number E-MTAB-8036. All codes to reproduce the bioinformatics analysis are freely available from https://github.com/CTR-BFX/2019-Menelaou-Watson.

### Quantitative reverse transcription-PCR (qRT-PCR)

Primers were designed using NCBI Primer-BLAST software (Ye *et al.* 2012). For primer sequences, refer to Supplementary Table 1 (see section on supplementary materials given at the end of this article). Reverse transcription reactions were performed with the RevertAid H Minus reverse transcriptase (Thermo Scientific) and random hexamer primers (Thermo Scientific) using 1 μg of total RNA according to manufacturer’s instructions. PCR amplification was performed using MESA SYBR Green qPCR MasterMix Plus (Eurogentec, Liege, Belgium) on a DNA Engine Opticon2 thermocycler (BioRad). Transcript levels were normalised to *Polr2a* RNA and analysed using the comparative Ct method. cDNA levels in C57Bl/6 control placentas were normalised to 1. For qRT-PCR validation of the RNA-seq experiment, 4–7 whole male or female placentas were analysed per experimental group from 2 to 4 litters and were independent of those assessed by RNA-seq. Experiments were conducted in technical triplicates.

### Bisulfite pyrosequencing

Genomic DNA was bisulphite converted using the Imprint DNA Modification Kit (Sigma-Aldrich) using the one-step modification procedure. Pyrosequencing assays were designed using PyroMark Assay Design SW 2.0 software (QIAGEN). For primer sequences, refer to Supplementary Table 1. PCR was performed in triplicate using HotStarTaq DNA polymerase (QIAGEN) and 5 ng of bisulphite-converted DNA with the following PCR conditions: 95°C for 5 min followed by 95°C for 30 s, 56°C for 30 s and 72°C for 55 s for 40 cycles, then 72°C for 5 min. PCR products were bound to Streptavidin Sepharose High-Performance beads (GE Healthcare) and purified by sequential washing in 70% ethanol, 0.4 mol/L NaOH and 10 mmol/L Tris-acetate (pH 7.6) using a Pyromark Q96 Vacuum Prep Workstation (QIAGEN). The purified product was mixed with the pyrosequencing primer in annealing buffer (20 mmol/L Tris-acetate pH 7.6, 2 mmol/L magnesium acetate), incubated at 85°C for 4 min and then at room temperature for 4 min. Pyrosequencing was conducted using PyroMark Gold reagents (QIAGEN) on a PyroMark MD pyrosequencer (QIAGEN). Analysis of methylation status was performed using Pyro Q-CpG 1.0.9 software (Biotage, Hengoed, UK). The mean CpG methylation was calculated using five whole female placentas from 3 to 4 litters and at least two technical replicates per experimental group.

### Statistical analysis

Statistical analyses were performed using GraphPad Prism 6 software. Parametric data (e.g., litter sizes, crown-rump lengths, placenta weights, weight ratios) were analysed using *t* tests. Relative risks were determined to compare males and females of each phenotypic group, and *P* values were calculated using Fisher’s exact test. For the RNA-sequencing data, the nbinomTest was used in R to calculate the *P* value and *P*-adjusted value of differentially expressed genes (DEGs). For mRNA expression, data were analysed using Mann–Whitney tests or *t* tests with Welch correction where appropriate. Pyrosequencing data were analysed using *t* tests when primers spanned a single CpG site and two-way ANOVA with Sidak’s multiple comparisons test when primers spanned two or more CpG sites. Frequency of CpG repeats was determined using Fisher’s exact test. *P* < 0.05 was considered significant.

### Software and online resources

Graphs were generated using GraphPad Prism 6 software. DEGs were subject to enrichment analysis using Enrichr (http://amp.pharm.mssm.edu/Enrichr/) (Chen *et al.* 2013, Kuleshov *et al.* 2016). Placental gene expression was determined using the Mouse Encode Project (http://www.mouseencode.org/) (Yue *et al.* 2014) and Mouse Cell Atlas (http://bis.zju.edu.cn/MCA/) (Han *et al.* 2018). Some phenotype data were obtained from Deciphering the Mechanisms of Developmental Disorders database (https://dmdd.org.uk), which is funded by the Wellcome Trust (www.wellcome.ac.uk) with support from the Francis Crick Institute (www.crick.ac.uk) and is licensed under a Creative Commons Attribution license (https://creativecommons.org/licenses/by/4.0/legalcode). CpG islands were identified with the University of California, Santa Cruz (UCSC) Mouse Genome Browser (http://genome.ucsc.edu/) using NCBI37/mm9 mouse genome assembly of the C57Bl/6 genome (Haeussler *et al.* 2019). CpG island tracks were defined as a stretch of DNA between 200 and 1300 bp with a GC content >50% and a ratio of observed:expected CpG dinucleotides >0.6. The enhancer-promoter units (EPUs) were generated according to previously published data (Shen *et al.* 2012). For this analysis, UCSC Mouse Genome Browser (http://genome.ucsc.edu/) using NCBI37/mm9 mouse genome assembly of the C57Bl/6 genome (Haeussler *et al.* 2019) was used to determine CpG island tracks and to analyse C57Bl/6 placenta histone modification peaks from the ENCODE ChIP-seq data set (GEO accession: GSM1000133, GSM1000134) (ENCODE Project Consortium 2012, Yue *et al.* 2014). The NCBI37/mm9 coordinates were lifted over to GRCm38/mm10 using LiftOver tool from UCSC. To generate an intersect between DEGs (log2fc1) and EPUs, bedtools2 (v2.26.0) software was used. Random gene lists were generated using Random Gene Set Generator (www.molbiotools.com).

## Results

### Blastocyst transfer potentially leads to increased placental efficiency at E10.5

To better understand how blastocyst transfer affects foeto-placental development, we mated C57Bl/6 female mice (*n* = 10) without superovulation to C57Bl/6 males (*n* = 10). Pre-implantation embryos were flushed at E3.25 from oviducts and uteri of donor females and transferred into oviducts of pseudopregnant B6D2F1 females. Transferred blastocysts were allowed to implant and were dissected at E10.5 (staged according to recipient females (Ueda *et al.* 2003)) for phenotyping. C57Bl/6 control conceptuses were derived from natural matings, did not undergo the blastocyst transfer procedure, and were dissected at E10.5 for similar analyses.

**Figure 1:**
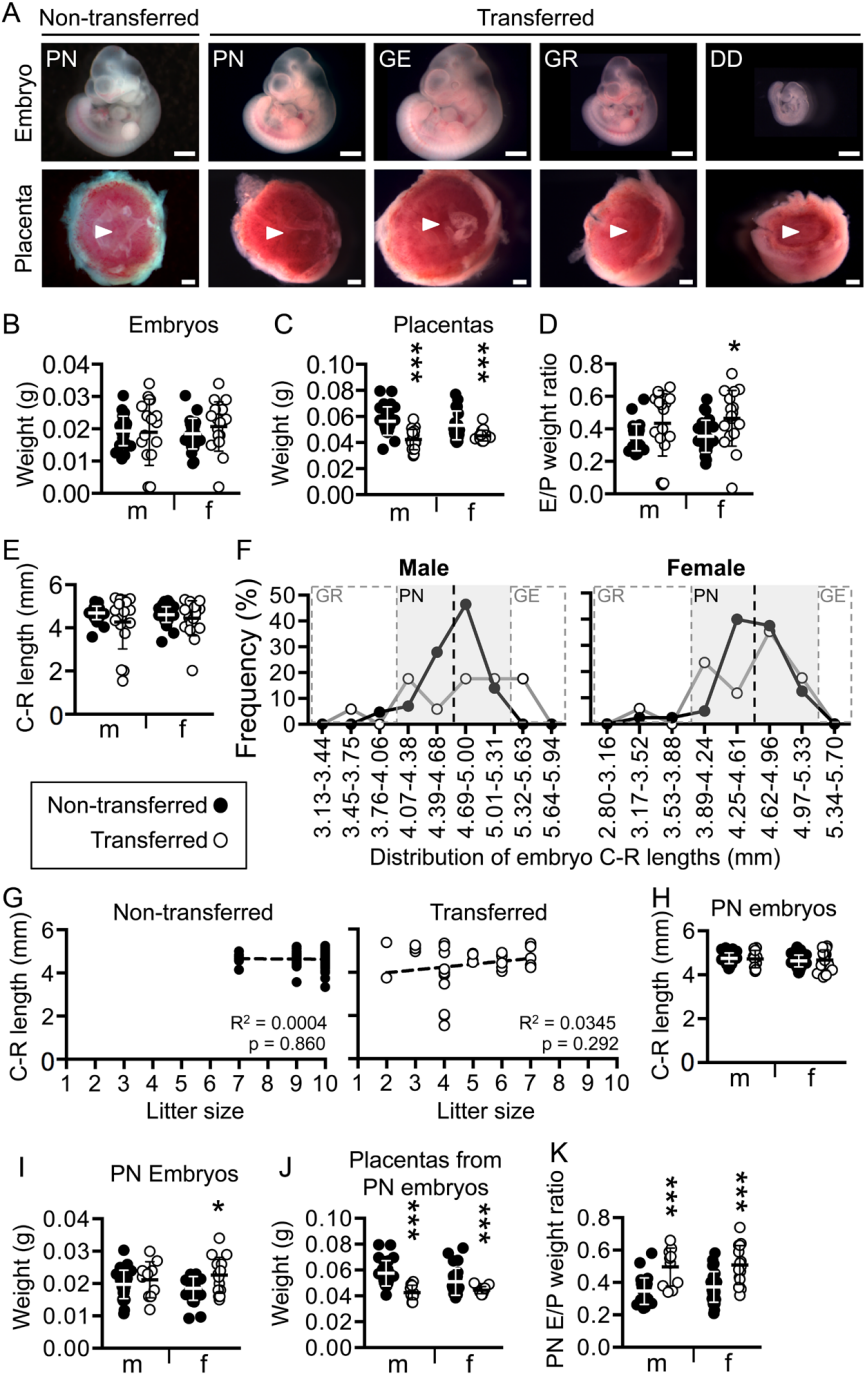
Blastocyst transfer in mice results in small placentas with a potential for increased efficiency at E10.5. (A) Images of embryos and placentas from non-transferred and transferred conceptuses at E10.5. Embryonic phenotypes shown include phenotypically normal (PN), growth enhanced (GE), growth restricted (GR), and developmentally delayed (DD) as determined by crown-rump lengths and somite pair counts. Arrowhead indicates where the allantois (i.e. the umbilical cord) was attached to the placenta. Scale bars: 500 μm. (B, C, D and E) Graphs showing (B) embryo weights, (C) placenta weights, (D) embryo:placenta (E/P) weight ratios, and (E) embryo crown-rump (CR) lengths in all non-transferred (black circles) and transferred (white circles) conceptuses at E10.5. Values are shown for male (m) and female (f) conceptuses (*n* = 17–43) and data are presented as mean ± s.d. Independent *t* test, *P < 0.05, ***P < 0.001. (F) Frequency distribution curves of embryo CR lengths as determined by sex for non-transferred (black solid line) and transferred (grey solid line) embryos. Only embryos staged as E10.5 were considered. Black dotted lines indicate the mean crown-rump length for non-transferred embryos. Lengths that fall within the grey shading indicate conceptuses that are grossly phenotypically normal (PN). Grey dotted lines indicate two s.d.s from the control mean. Crown-rump lengths that were greater than two s.d.s below the mean were considered GR and greater than two s.d.s above the mean were considered GE. (G) Linear regression analysis of litter size versus embryo CR length in control (black circles) and transferred (white circles) conceptuses. A line of best fit is indicated (dotted line). (H, I, J and K) Graphs showing parameters for PN conceptuses only including (H) embryo CR length, (I) embryo weight, (J) placenta weight, and (K) E/P weight ratios in non-transferred (black circles) and transferred (white circles) conceptuses. Data are presented as mean ± s.d. and is shown for male (m) and female (f) conceptuses (*n* = 10–43 conceptuses). Independent *t* tests, *P* < 0.05, ***P < 0.001.

Prior to transfer, litter sizes at E3.25 ranged between 7 and 10 embryos and included compact morula- and blastocyst-staged embryos (Table 1). Two litters contained at least one embryo with an abnormal appearance (Table 1). However, all embryos were transferred regardless of appearance. Ten pseudopregnant B6D2F1 females received embryos but only eight were pregnant at E10.5 (80% efficiency). The average implantation rate was 47.2% when all transferred litters were considered and 58.9% when only considering transfers that resulted in pregnancy. Thus, litter sizes in the blastocyst transfer group were significantly smaller (5.4 ± 0.5 implantation sites/litter; *P* < 0.001) than control litters (9.5 ± 0.4 implantation sites/litter) (Table 2). No congenital malformations or gross placental phenotypes were apparent in control or transferred conceptuses at E10.5 (see Methods, Table 2). However, one litter in the transfer group was developmentally delayed since the number of somite pairs in these embryos ranged between 16 and 22 pairs (Fig. 1A) instead of the expected 30–40 somite pairs. At E10.5, 20.9% of the transferred conceptuses were resorbed compared to only 5.3% of controls (Table 2). Although not statistically significant (*P* = 0.200), this result suggested that blastocyst transfer might lead to increased post-implantation lethality before E10.5. A larger data set is required to explore this finding further.

**Table 1:** Embryonic phenotypes before and after blastocyst transfer in mice.

Recipient female ID	Phenotype at E3.5 before transfer				No. of conceptuses at E10.5 (implantation rate (%))	Phenotype at E10.5 after transfer					
	No. of embryos transferred	Blastocyst stage	Morula stage	Abnormal		PN	GE	GR	DD	CM	R
C57-T1	10	5	4	1	6 (60.0%)	3	1	0	0	0	2
C57-T2	9	6	3	0	0	–	–	–	–	–	–
C57-T3	10	3	7	0	6 (60.0%)	5	0	0	0	0	1
C57-T4	8	4	4	0	4 (50.0%)	0	0	0	4	0	0
C57-T5	10	4	3	3	6 (60.0%)	6	0	0	0	0	0
C57-T6	9	7	2	0	3 (33.3%)	0	1	1	0	0	1
C57-T7	8	7	1	0	5 (71.4%)	3	0	1	0	0	1
C57-T8	9	5	4	0	0	–	–	–	–	–	–
C57-T9	8	7	1	0	7 (87.5%)	5	1	0	0	0	1
C57-T10	10	6	4	0	6 (60.0%)	3	0	0	0	0	3
Total	91	48	29	4	43 (47.2%)	25	3	2	4	0	9

CM, congenital malformation; DD, developmentally delayed; E, embryonic day; GE, growth enhanced; GR, growth restricted; PN, phenotypically normal; R, resorption.

**Table 2:** Phenotypic comparison of non-transferred and transferred mouse conceptuses at E10.5.

	C57Bl/6 non-transferred^*^	C57Bl/6 transferred^*^
No. of conceptuses assessed at E10.5		
Total	95 (10 litters)	43 (8 litters)
Male	44^†^	17^†^
Female	45^†^	17^†^
Average no. of conceptuses/litter		
Total	9.5 ± 0.4	5.4 ± 0.5***
Male	4.4 ± 0.5 (46.5%)^†^	2.1 ± 0.5 (39.5%)^†^
Female	4.5 ± 0.4 (47.4%)^†^	2.1 ± 0.5 (39.5%)^†^
Phenotypes		
Phenotypically normal		
Total	8.6 ± 0.5 (90.5%)	3.1 ± 0.8*** (58.1%)
Male	4.2 ± 0.5 (95.5%)	1.3 ± 0.5*** (58.8%)
Female	4.1 ± 0.4 (91.1%)	1.9 ± 0.5*** (88.2%)
Growth enhanced		
Total	0.0	0.4 ± 0.2^‡^ (7.0%)
Male	0.0	0.4 ± 0.2^‡^ (17.6%)
Female	0.0	0.0
Growth restricted		
Total	0.4 ± 0.2 (4.2%)	0.3 ± 0.2 (4.7%)
Male	0.2 ± 0.1 (4.5%)	0.1 ± 0.1 (5.9%)
Female	0.2 ± 0.1 (4.4%)	0.1 ± 0.1 (5.9%)
Developmental delay		
Total	0.0	0.5 ± 0.5 (9.3%)
Male	0.0	0.4 ± 0.4 (17.6%)
Female	0.0	0.1 ± 0.1 (5.9%)
Congenital malformations		
Total	0.0	0.0
Male	0.0	0.0
Female	0.0	0.0
Resorptions^†^		
Total	0.5 ± 0.3 (5.3%)	1.1 ± 0.4 (20.9%)
Male	–	–
Female	–	–

*Data are presented as average number of conceptuses (±s.e.) per litter unless otherwise indicated. Number in brackets indicates the percentage of total conceptuses with each phenotype. ^†^Resorptions could not be genotyped for sex due to maternal tissue contamination. ^‡^*P* = 0.08.

Overall, male and female conceptuses were present in a 1:1 ratio at E10.5 in both control and transferred groups (Table 2) indicating that sex was not a selective factor on survival after blastocyst transfer. While average embryo weights were similar (Fig. 1B), placental weights were lower in transferred conceptuses compared to controls (Fig. 1C) suggesting that the blastocyst transfer protocol might alter placental development. Furthermore, embryo:placenta weight ratios were higher in transferred conceptuses; yet, only significantly so in transferred female conceptuses (*P* = 0.025; Fig. 1D).

To further define the growth phenotype, embryo crown-rump lengths were measured. While mean lengths were not significantly different between control and transfer groups (Fig. 1E), frequency distribution curves of crown-rump lengths were generated (Fig. 1F) to detect specific embryos with abnormal growth. Defective growth was defined as crown-rump lengths that were >2 s.d. from the mean length of control embryos. Regardless of sex, a similar frequency of growth restriction was observed in control (4.4–4.5% of embryos) and transferred conceptuses at E10.5 (5.9% of embryos, *P* = 0.687; Fig. 1A, F and Table 2). Interestingly, 3 out of 17 (17.6%) of the transferred male embryos displayed growth enhancement (Fig. 1A, F and Table 2). This frequency was not statistically significant (*P* = 0.08), likely due to the small sample size. No transferred female embryos or control embryos were growth enhanced (Fig. 1F and Table 2) suggesting that this phenotype might be sexually dimorphic. A lack of correlation between litter size and crown-rump length in control or transferred litters at E10.5 (Fig. 1G) suggested that litter size is an unlikely confounder of this study.

When all phenotypes (e.g., growth restriction, growth enhancement, developmental delay) and resorptions were grouped together, fewer embryos were classified as ‘phenotypically normal’ (PN) in the transferred group (55.8%; *P* < 0.001) compared to controls (91.6%; Table 2). When the analysis was restricted to conceptuses with PN embryos (Fig. 1A and F), average weight of the associated placentas remained lower in transferred conceptuses compared to controls, regardless of sex (*P* < 0.004; Fig. 1J). Despite this, average embryo weight was normal in PN males (*P* = 0.472) and higher in PN females (*P* = 0.019) compared to same-sex controls (Fig. 1I). These data suggested that the placentas of transferred conceptuses, though small, were potentially more efficient in both sexes. This hypothesis was reinforced by increased embryo:placenta weight ratios in PN transferred conceptuses (*P* < 0.002; Fig. 1K). Future histological and functional analyses are required to confirm this hypothesis. Altogether, these data imply that after blastocyst transfer, normal growth trajectory of the embryo is likely reliant upon functional compensation by the placenta.

### Blastocyst transfer alters the placental transcriptome at E10.5

**Figure 2:**
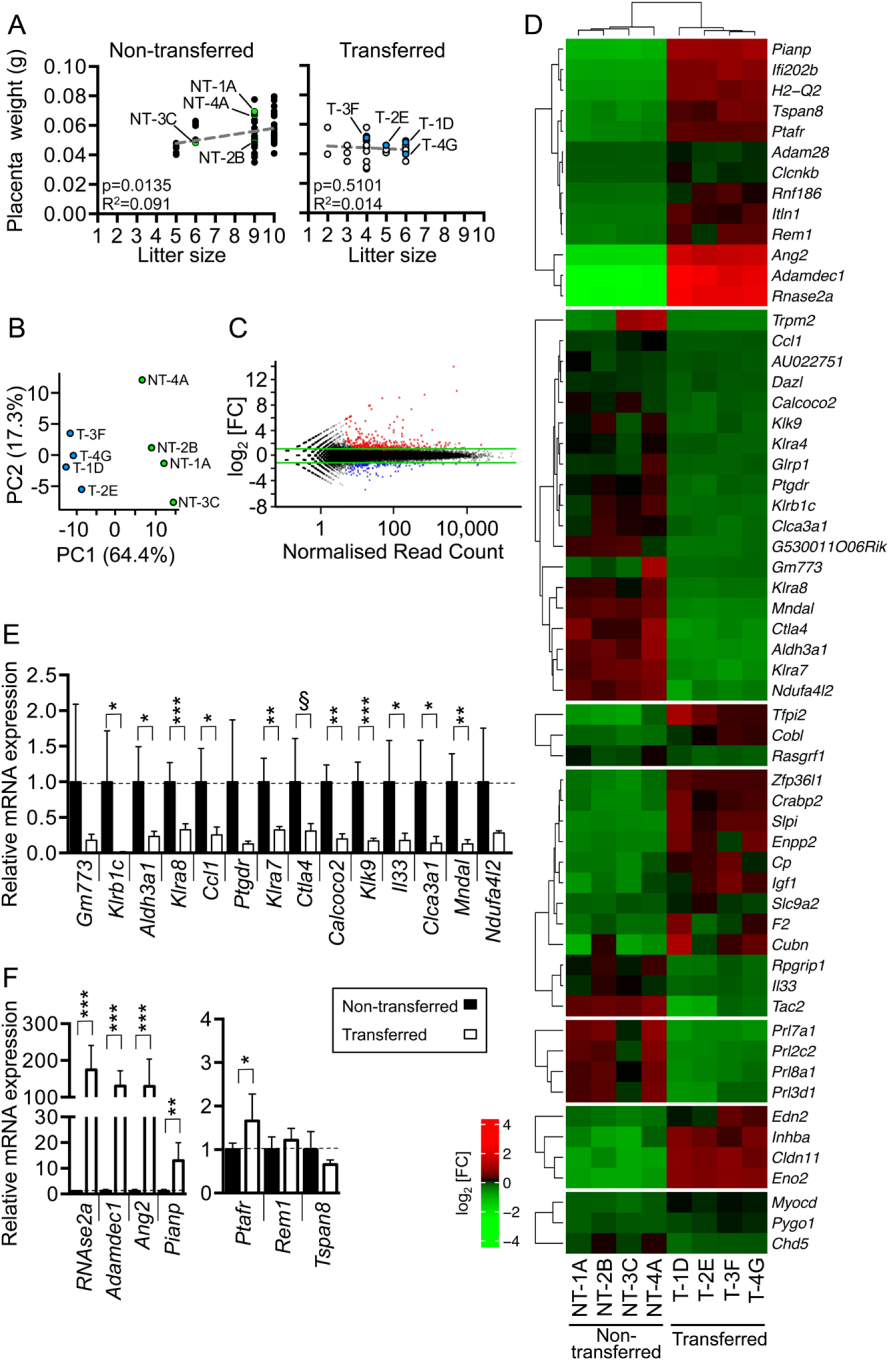
Blastocyst transfer causes differential gene expression in mouse placentas at E10.5. An RNA-seq analysis was performed on whole male placentas from non-transferred and transferred experimental groups. *n* = 4 placentas from 3 to 4 litters per group were analysed. Placentas assessed via RNA-seq are labeled in panels A, B, C and D with the placenta ID (non-transferred (NT) or transferred (T) followed by the sample number and letter ID indicating the litter). (A) Graphs plotting litter size versus placental weight of male and female non-transferred placentas (left panel) and transferred placentas (right panel). The line of best fit (grey dotted line) is indicated, and the *P* and *R*^2^ values from a linear regression analysis are shown. Green and blue data points are samples used in the RNA-seq experiment. (B) Principal component analysis of the RNA-seq data of whole male placentas from control non-transferred (green circles) and transferred (blue circles) conceptuses. (C) MA plot revealing 543 differentially expressed genes (DEGs) with a fold change > log_2_1.0 (i.e., fold change ≥ 2; *P* < 0.05) in placentas from transferred conceptuses compared to non-transferred controls. 188 DEGs were downregulated (blue) and 355 DEGs were upregulated (red). (D) Heat map showing differential expression of selected DEGs in non-transferred (NT) and transferred (T) placentas. Scale shown is log_2_(fold change). Red, gene upregulation; green, gene downregulation. (E and F) Validation of RNA-seq data by qRT-PCR analysis in independent placentas from non-transferred (black bars) and transferred (white bars) conceptuses. Data are presented as fold change compared to controls (normalised to 1). *n* = 4–7 placentas per group from 2 to 4 litters. Graphs show qRT-PCR data of representative genes that were significantly (E) downregulated or (F) upregulated in transferred placentas. Unpaired *t* tests with Welch correction or Mann–Whitney tests where appropriate, ^§^*P* = 0.05, *P < 0.05, **P < 0.01, ***P < 0.001.

Typically, large litters result in smaller conceptuses, particularly in late gestation when competition for maternal resources is highest (Ishikawa *et al.* 2006). In our data at E10.5, we observed that neither embryo crown-rump length (Fig. 1G) nor placenta weight (Fig. 2A) correlated with litter size. These data further demonstrate that litter size is an unlikely confounder in our study.

We sought to determine the effect of blastocyst transfer on the placental transcriptome. A genome-wide transcriptome analysis was performed via RNA sequencing to determine differential mRNA expression in whole placentas from control (*n* = 4) or transferred (*n* = 4) C57Bl/6 conceptuses at E10.5. Since the placenta transcriptome is known to exhibit some sexual dimorphism (Gonzalez *et al.* 2018) and due to tissue availability, only male placentas associated with PN embryos were selected for analysis. cDNA libraries generated from placental RNA were sequenced using the NextSeq500 (Illumina) platform and bioinformatically analysed.

A principal component analysis revealed that, based on RNA content, the placentas of control and transferred conceptuses clustered separately (Fig. 2B). Differential mRNA expression was determined when *log*_2_(fold change) > 1 (i.e. fold change ≥ 2; *P* < 0.05). Remarkably, 543 genes were differentially expressed in transferred placentas compared to controls including 355 upregulated and 188 downregulated genes (Fig. 2C and D). We selected 27 (5.0%) DEGs for validation via qRT-PCR using RNA isolated from independent whole male placentas (*n* = 4–7 placentas/group) (Figs 2E, F and 4B). Thirteen out of 17 genes that were downregulated in the RNA-sequencing experiment were also downregulated in the qRT-PCR analysis (*P* < 0.05), with the remaining four genes showing a strong downward trend (Figs 2E and 4B). Furthermore, eight out of ten upregulated genes were also statistically validated by qRT-PCR (*P* < 0.05; Figs 2F and 4B). Therefore, the RNA-sequencing experiment was deemed robust and reliable.

### All major placental cell types represented by differentially expressed genes

To determine if blastocyst transfer affected gene expression in specific placental cell types, we performed an extensive literature and database search to resolve the spatial expression of the DEGs in mouse placentation sites. The primary resource for this analysis was a published single-cell RNA sequencing data set on whole C57Bl/6 mouse placentas at E14.5 (Han *et al.* 2018). Although the developmental stage assessed in Han *et al.* (2018) differed slightly from our analysis, the data can be used as a predictive indicator of spatial expression.

**Figure 3:**
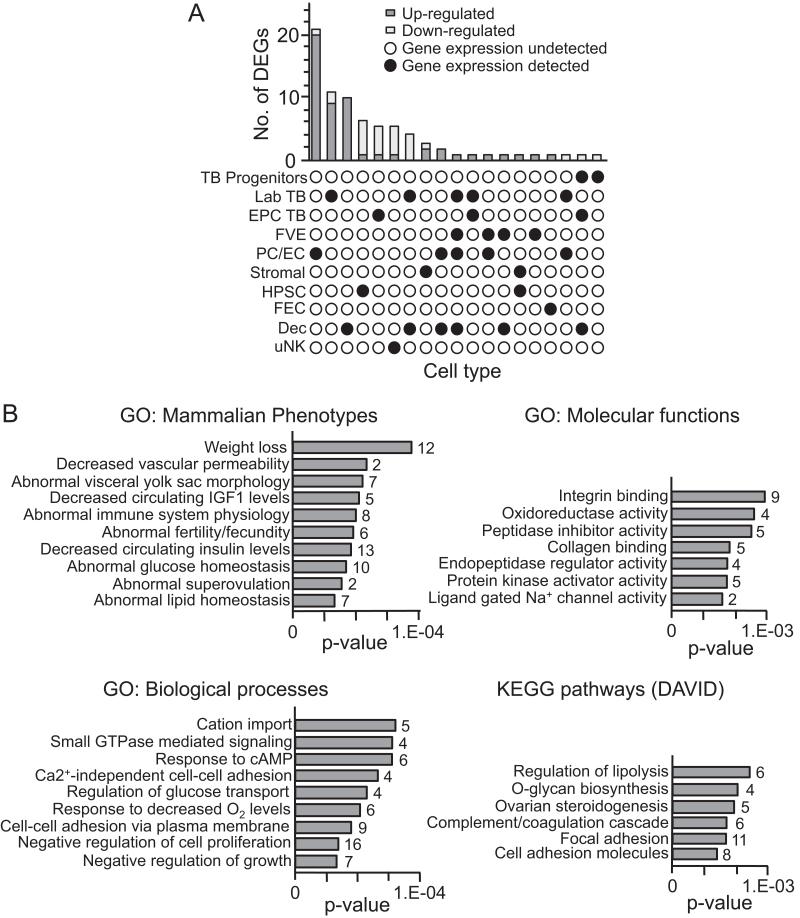
Spatial expression and ontological analyses of DEGs identified in placentas of transferred mouse conceptuses at E10.5. (A) UpsetR plot summarizing the predicted spatial expression of 79 DEGs in the mouse placentation site. Data were largely obtained from a published single-cell RNA sequencing data set on whole mouse placenta at E14.5 (Han *et al.* 2018). See also Supplementary Table 3. Dark grey bars, upregulated DEGs; light grey bars, downregulated DEGs; black circles indicate cell-specific expression; white circle indicates that expression was undetected. TB, trophoblast; Lab TB, labyrinth trophoblast; EPC TB, ectoplacental cone trophoblast; FVE, fetal endothelial vascular cells; PE/EC, parietal endoderm or endodermal cells; HPSC, haematopoietic stem cells; FEC, foetal erythroid cell; Dec, decidua; uNK, uterine natural killer cell. (B) Gene ontology term enrichment analysis for DEGs in placentas of transferred conceptuses. The numbers of genes in each term are also given.

Of the 543 DEGs that were identified, placental expression of the majority of DEGs (362 genes) is not currently described in mouse. Expression of 102 DEGs has been reported in the C57Bl/6 mouse placenta at term but without information about cell type specificity (Yue *et al.* 2014) (Supplementary Table 2). Spatial expression of the remaining 79 genes has been characterized. In several cases (14 DEGs), gene expression occurred in more than one placental cell population (Fig. 3A and Supplementary Table 3). Additionally, most cell types present in the mouse placentation site were represented by DEGs (e.g., trophoblast, endothelial, endoderm, stromal, decidual, hematopoietic lineages) (Fig. 3A and Supplementary Table 3) further indicating that blastocyst transfer is unlikely to affect a single cell lineage. A similar proportion of DEGs were detected in trophoblast cells (26/79 DEGs, 32.9%) and endodermal cells (27/79 DEGs, 34.2%), though fewer DEGs were detected in the decidua (19/79 DEGs, 24.1%) and foetal vascular endothelium (5/79, DEGs, 6.3%) (Fig. 3A and Supplementary Table 3). It will be important in the future to validate the spatial expression of these genes in the mouse placentation site at E10.5 using *in situ* hybridisation. Overall, this result suggested that blastocyst transfer caused broad transcriptional changes throughout the mouse placentation site at midgestation even when the embryo was considered phenotypically normal.

### DEGs were enriched for genes associated with placental growth and function

The primary annotation terms enriched in placentas from transferred conceptuses at E10.5 included genes important for placental development, growth and function (Fig. 3B). Enrichment groups included genes associated with specific mammalian phenotypes (e.g., decreased vascular permeability, abnormal visceral yolk sac, abnormal lipid and glucose homeostasis, and decreased circulating IGF1 and insulin levels), and genes required for specific biological processes (e.g., cell adhesion, response to decreased oxygen levels, and negative regulation of cell proliferation and growth) (Fig. 3B). At least 13 DEGs identified are important for placental development and/or function as evidenced by gene knockout and overexpression studies (Table 3). Furthermore, 31 DEGs encode for proteins with nutrient transporter activity (24/31 (77.4%) DEGs were upregulated; 7/31 (22.6%) DEGs were downregulated) including gap junction protein 1 (*Gjb1*), and those important for lipid and fatty acid transport (*Slc27a2*, *Mttp*, *Apom*, *Amn*) and glutamate transport (*Slc17a8*, *Slc7a11*) (Table 4). Together, these data supported the hypothesis that blastocyst transfer results in structural and functional compensation by the placenta with the aim of maintaining embryo growth. Whether changes in gene expression lead to altered protein expression or activity in transferred placentas requires exploration.

**Table 3:** Differentially expressed genes in placentas of transferred mouse conceptuses at E10.5 that are implicated in growth, placental phenotypes, and/or embryonic lethality.

Gene	Gene function	Mouse-knockout phenotype or clinical characteristics (timing of embryonic lethality)	Reference	FC
Genetic knockout or decreased expression: defective labyrinth, placental insufficiency and/or FGR				
*Cubn*	Receptor-mediated endocytosis	Defects in chorioallantoic attachment (E10.5)	(Smith *et al.* 2006)	2.9
*Enpp2*	Angiogenesis	Defective chorioallantoic attachment (E10.5)	(Fotopoulou *et al.* 2010)	2.5
*Zfp36l1*	RNA-binding protein	Failure of chorioallantoic attachment^†^, reduced branching morphogenesis in labyrinth layer, small spongiotrophoblast layer (E10.5^†^)	(Stumpo *et al.* 2004, Bell *et al.* 2006)	2.1
*F2*	Maintenance of vascular integrity	Reduced or absent branching morphogenesis in labyrinth layer (E10.5^†^)	(Sun *et al.* 1998, Xue *et al.* 1998)	3.4
*Rpgrip1*	Regulator of ciliary protein traffic	Abnormal vasculature in labyrinth at E14.5 (‘Pre-weaning’ lethality)	(Perez-Garcia *et al.* 2018)	−2.0
*Slpi*	Serine protease inhibitor	Downregulated expression in rat model of placental insufficiency (ND)	(Goyal *et al.* 2010)	2.5
*Igf1*	Insulin-like growth factor	Severely growth restricted; unknown placental phenotype (Perinatal)	(Liu & LeRoith 1999)	2.2
Increased gene expression: trophoblast phenotype, placental insufficiency, preeclampsia, or FGR				
*Aldh3a1*	Aldehyde dehydrogenase	OE in mouse TSCs prevents differentiation into *Tpbpa+* cells (EPC lineage) (ND)	(Nishiyama *et al.* 2015)	−9.3
*Il33*	Interleukin	Inhibits trophoblast invasion and adhesion* in vitro* (ND)	(Wang *et al.* 2017)	−4.0
*Tac2*	Disintegrin and metalloproteinase	Fetal growth restriction in humans associated with increased placental expression (ND)	(Ozler *et al.* 2016)	−3.1
*Crabp2*	Retinoic acid signalling pathway	OE in endometrial cell line causes decreased proliferation of trophoblast spheroids (ND)	(Lee *et al.* 2011)	2.3
*Cp*	Iron peroxidase	Increased placental expression associated with pre-eclampsia in humans (ND)	(Guller *et al.* 2008)	2.6
*Slc9a2*	Sodium/hydrogen exchanger	Upregulated in rat model of placental insufficiency; unknown placental phenotype (possible embryonic lethality^†^)	(Goyal *et al.* 2010)	2.9

^†^Incomplete penetrance.

E, embryonic day; EPC, ectoplacental cone; FC, fold change in RNA-sequencing experiment; FGR, foetal growth restriction; ND, not determined; OE, overexpression; TSCs, trophoblast stem cells.

**Table 4:** Differentially expressed genes in placentas of transferred mouse conceptuses at E10.5 that encode for proteins with transporter function.

Gene	Fold change	Gene function
Downregulated genes		
*Trpm2*	−10.1	Calcium channel
*Slc28a2l (Gm14085)*	−5.15	Purine nucleoside transporter
*Clca3a1*	−3.90	Calcium-activated chloride channel
*Slc17a8*	−3.00	l-glutamate transporter
*Ndufa4l2*	−2.93	NADH dehydrogenase (ubiquinone), mitochondria
*Slc28a2*	−2.03	Sodium-coupled purine nucleoside transporter
*Kcnab2*	−2.03	Voltage-gated potassium channel, NADH oxidation
Upregulated genes		
*Kcnd2*	5.13	Voltage-gated potassium channel
*Kcne3*	3.91	Voltage-gated potassium channel
*Kcnj12*	3.07	ATP-sensitive inward rectifier potassium channel
*Atp6v1c2*	2.89	Proton-exporting ATPase, phosphorylative mechanism
*Slc9a2*	2.87	Sodium/hydrogen exchanger
*Slc13a5*	2.87	Sodium-dependent citrate transporter
*Gjb1*	2.84	Gap junction protein
*Slc7a9*	2.72	Sodium-independent l-cystine transporter
*Abcc6*	2.68	ATP-binding cassette transporter
*Cftr*	2.62	ATP-gated chloride channel
*Slc27a2*	2.60	Fatty acid transporter
*Hcn4*	2.58	Cyclic nucleotide-gated potassium channel
*Slc7a11*	2.57	Cysteine/glutamine transporter
*Cacna1b*	2.56	Voltage-gated calcium channel
*Jph2*	2.42	Calcium channel
*Slc39a8*	2.31	Zinc ion transporter
*Trpm3*	2.31	Cation channel
*Mttp*	2.30	Microsomal triglyceride transfer protein
*Kcnk2*	2.21	Potassium channel
*Asic2*	2.14	Voltage-gated sodium channel
*Apom*	2.09	Lipid transport
*Scnn1g*	2.07	Amiloride-sensitive sodium channel
*Amn*	2.04	Cobalamine and lipid transport
*Slc4a1*	2.03	Chloride/bicarbonate exchanger

### Dysregulation of DNA methylation at proximal promoter regions by blastocyst transfer is unlikely to disrupt transcription

**Figure 4:**
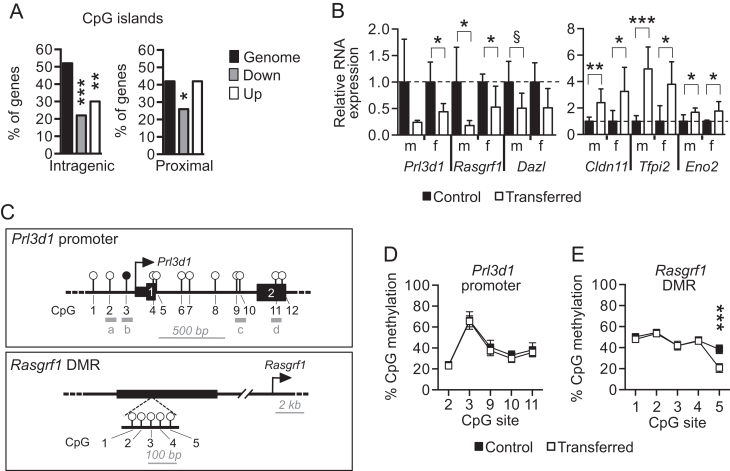
Alteration of proximal promoter CpG methylation might not disrupt transcriptional change in the mouse placenta after blastocyst transfer. (A) The percentage of the top 50 downregulated DEGs (grey bars, FC >3.1) or upregulated DEGs (white bars, FC >6.7) after blastocyst transfer that associate with intragenic or proximal promoter CpG repeats (within 20 kb region). The percentage of 50 randomly selected genes is indicated as a proxy for the genome (black bars). Fisher’s exact test, *P < 0.05, **P < 0.01, ***P < 0.001. (B) Validation of differential gene expression in male (m) and female (f) placentas from non-transferred (black bars) and transferred (white bars) conceptuses at E10.5. Data are presented as fold change compared to controls (normalised to 1; mean ± s.d.). *n* = 4–7 placentas/group from 3 to 4 litters. Independent *t* test. ^§^*P* = 0.05; *P < 0.05; **P < 0.01; ***P < 0.001. (C) Schematic drawings of the *Prl3d1* promoter and *Rasgrf1* differentially methylated region (DMR). Modified from (Yoon *et al.* 2005, Hayakawa *et al.* 2012). Open and closed circles represent unmethylated and methylated CpG sites in placental tissue, respectively. a–d represent regions assessed by bisulfite pyrosequencing in *Prl3d1* promoter. (D and E) CpG site-specific methylation (mean ± s.e.) in (D) the promoter region of the *Prl3d1* gene and (E) the *Rasgrf1* DMR as determined via bisulfite pyrosequencing of DNA from control (black squares) and transferred (white squares) female placentas at E10.5. *n* = 5 placentas/group from 3 to 4 litters. Independent *t* tests when primers spanned a single CpG site (i.e. *Prl3d1*, regions a, b, d) and two-way ANOVA with Sidak’s multiple comparisons test when primers spanned two or more CpG sites (i.e. *Prl3d1*, region c; *Rasgrf1* DMR). ***P < 0.001.

The mechanism by which blastocyst transfer alters gene expression is not well understood, though epigenetic dysregulation has been implicated (Choux *et al.* 2015, Canovas *et al.* 2017). To examine the potential sensitivity of the DEGs to changes in DNA methylation, we first evaluated whether the top 50 downregulated genes (fold change >3.1) and top 50 upregulated genes (fold change >6.7) contained intragenic CpG islands or proximal promoter CpG islands (within 20 kb of the gene) (Fig. 4A). This result was compared to 50 randomly selected genes as a proxy for the rest of the genome. CpG islands are usually associated with methylation and are known to regulate gene expression (Gardiner-Garden & Frommer 1987). We identified CpG islands using the CpG island track within the UCSC Mouse Genome Browser (Haeussler *et al.* 2019; see Methods). While this initial analysis did not determine whether the CpG islands were methylated or directly regulate gene expression, it highlighted the potential for gene dysregulation when methylation patterns are altered. Compared to the genome, both up and downregulated gene sets were less likely to contain intragenic CpG islands (22–30% of DEGs compared of 55% of genes genome-wide, *P* < 0.005; Fig. 4A). Moreover, downregulated DEGs were less frequently associated with proximal promoter CpG islands (26% of DEGs, *P* = 0.025) than both upregulated DEGs and the genome (42% of genes; Fig. 4A). Overall, these findings suggested that genes that were differentially expressed after blastocyst transfer were less likely to be cis-regulated by intragenic or proximal promoter CpG methylation compared to the genome at large.

We identified 15 DEGs whose regulation was directly linked to CpG methylation at specific genomic locations by other studies (Table 5). Based on the published data and our RNA-seq results, we predicted that seven of these DEGs should be associated with CpG hypomethylation and eight DEGs with CpG hypermethylation (Table 5). We sought to test CpG methylation in two loci (e.g., *Prl3d1* promoter and the differentially methylated region (DMR) associated with the* Rasgrf1* gene) via bisulfite pyrosequencing. Due to tissue availability, bisulfite pyrosequencing was carried out on whole female placentas (*n* = 5 placentas from 3 to 4 litters/group), even though the RNA-sequencing experiment assessed whole male placentas. To rectify potential sex differences, we first verified that female placentas from transferred conceptuses showed similar dysregulation of DEGs as male transferred conceptuses by performing qRT-PCR analysis. In all six DEGs assessed (i.e., *Prl3d1*, *Rasgrf1*, *Dazl*, *Cldn11*, *Tfpi2*, *Eno2*), female and male transferred placentas showed similar patterns of gene misexpression relative to same-sex controls (Fig. 4B). Thus, regardless of sex, we expected CpG hypermethylation in the *Prl3d1* promoter (Hayakawa *et al.* 2012) (Fig. 4C) in transferred placentas since *Prl3d1* mRNA expression was low (Figs 2D and 4B). Nevertheless, five CpG sites across the *Prl3d1* promoter revealed comparable levels of methylation in non-transferred and transferred placenta groups (Fig. 4D). This methylation pattern was also similar to normal C57Bl/6N placentas without decidua at E14.5 (Hayakawa *et al.* 2012) indicating that the presence of decidua in our placentas was an unlikely confounder. Likewise, we assessed CpG methylation in the *Rasgrf1* DMR (Fig. 4C). Reduced *Rasgrf1* mRNA expression in transferred placentas (Figs 2D and 4B) intimated that hypomethylation of the *Rasgrf1* DMR was expected (Yoon *et al.* 2005). However, four out of five CpGs assessed showed similar levels of methylation in transferred and control placentas (Fig. 4E). One CpG site in the *Rasgrf1* DMR (CpG-5) was hypomethylated in transferred placentas (20.7 ± 4.1% methylated vs 38.6 ± 4.0% methylated in controls; *P* < 0.0001; Fig. 4E), but hypomethylation of a single CpG among many is unlikely to be responsible for increased *Rasgrf1* mRNA expression. Altogether, these data supported the hypothesis that disruption of DNA methylation at promoter regions by blastocyst transfer might not lead to transcriptional changes in the mouse placenta at E10.5.

**Table 5:** Differentially expressed genes in placentas from transferred mouse conceptuses at E10.5 with known regulation by DNA methylation.

Gene name	Function	Known characteristics of epigenetically regulated expression	FC	Predicted CpG methylation*	Ref.
Downregulated genes					
*Trpm2*	Cation channel	Methylation of inner CpG island: gene repression	−10.1	Hyper	Orfanelli *et al.* 2008
*Klra4*	Killer cell lectin-like receptor; cell adhesion	Promoter hypomethylation: gene activation	−8.8	Hyper	Rouhi *et al.* 2009
*Dazl*	RNA-binding protein	Promoter methylation: gene repression	−5.0	Hyper	Hackett *et al.* 2012
*Prl8a1*	Prolactin hormone family	Placenta-specific promoter hypomethylation: gene activation	−2.9	Hyper	Hayakawa *et al.* 2012
*Prl7a1*	Prolactin hormone family	Placenta-specific promoter hypomethylation: gene activation	−2.9	Hyper	Hayakawa *et al.* 2012
*Rasgrf1*	Guanine nucleotide-releasing factor	(Imprinted) Paternally inherited allele is methylated and biallelically expressed in the placenta; methylation leads to gene activation	−2.7	Hypo	Yoon *et al.* 2005, Dockery *et al.* 2009
*Kazald1*	Insulin growth factor-binding protein family	Promoter hypomethylation: gene activation	−2.3	Hyper	Wang *et al.* 2013*a*
*Prl3d1*	Prolactin hormone family	Placenta-specific promoter hypomethylation: gene activation	−2.0	Hyper	Hayakawa *et al.* 2012
Upregulated DEGs					
*Edn2*	Angiogenesis	Hypomethylation of intragenic region: gene activation	7.2	Hypo	Wang *et al.* 2013*b*
*Cldn11*	Gap junction protein	Promoter hypermethylation: gene repression	5.9	Hypo	Agarwal *et al.* 2009
*Eno2*	Glycolysis	Promoter hypermethylation: gene repression	5.7	Hypo	Wang *et al.* 2014
*Inhba*	TGFβ signalling pathway	Promoter hypermethylation in human placenta: gene repression	4.4	Hypo	Wilson *et al.* 2015
*Cfb*	Complement factor	Promoter hypermethylation: gene repression; in human placenta, promoter hypomethylated upon CytoT to SynT differentiation	4.4	Hypo	Yuen *et al.* 2013
*Tfpi2*	Serine protease	(Imprinted) Paternally inherited allele is methylated at the ICR, maternally inherited allele expressed the placenta	3.1	Hypo	Monk *et al.* 2008
*Cobl*	Actin interacting protein	(Imprinted) Tissue-specific parentally biased expression; methylation on maternally inherited allele at *Grb10* DMR in yolk sac results in *Cobl* expression from the maternal allele	2.6	Hyper	Shiura *et al.* 2009

*Predicted change in CpG methylation based on differential expression observed in RNA-sequencing experiment and published data.

CytoT, cytotrophoblast; FC, fold change; hyper, hypermethylated; hypo, hypomethylated; ICR, imprinting control region; SynT, syncytiotrophoblast.

### Some DEGs cluster in the genome implying common transcriptional regulation

**Figure 5:**
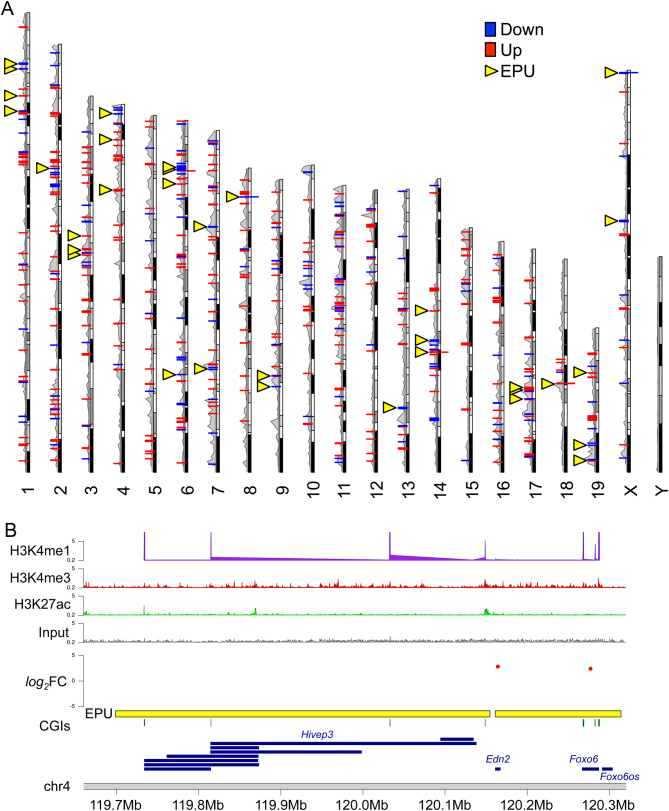
Clustering of differentially expressed genes (DEGs) in the mouse genome suggests shared long-range, cis-acting regulatory regions. (A) Schematic karyoplot representing mouse chromosomes 1–19, X and Y indicating the location of DEGs (log_2_(fold change) > 1) in whole placentas after embryo transfer as determined via RNA sequencing. Red, upregulated genes; blue, downregulated genes. Peaks on the left-hand side of each chromosome represent the degree of gene density in that genomic region. Yellow arrowheads indicate enhancer-promoter units (EPUs) containing at least two DEGs. (B, C and D) A schematic representation of two neighbouring EPUs (yellow bars) on chromosome 4 including one EPU (right) that contains the DEGs *Edn2* and *Foxo6*. The level of misexpression of these genes is indicated by the respective red dots on a *log*_2_(fold change) scale. Additional features include chromosomal location of peaks of enrichment for placental histone modifications, such as H3K4me1 (purple peaks), H3K4me3 (red peaks), and H3K27ac (light green peaks) generated using published ChIP-seq data (Yue *et al.* 2014), CpG islands (CGIs; green lines) and genes (blue). Grey peaks indicate ChIP-seq input. See also Supplementary Table 4.

Next, we determined the chromosomal location of DEGs (fold change >2) including 355 upregulated and 188 downregulated genes (Fig. 5A). While the DEGs were distributed throughout the genome, we observed several genomic clusters of two or more DEGs (Fig. 5A). Gene clustering was unlikely to occur by chance because no clusters were identified in a group of 60 randomly selected genes. This observation suggested that some DEGs might share common long-range, cis-acting regulatory mechanism(s). To explore this hypothesis further, we analysed a published data-set that defined enhancer-promoter units (EPUs) in C57Bl/6 placentas at term based on an active chromatin state at enhancers (i.e., enrichment for H3K4 monomethylation (me1) and H3K27 acetylation (ac)) and occupancy of RNA polymerase II at promoters (Shen *et al.* 2012). From these data, we predicted DEGs (fold change >2) that were coordinately regulated by cis-acting elements in the placenta based on their EPU co-localisation. A total of 33 EPUs were identified containing at least two DEGs and a maximum of eight DEGs (mean (±s.d.): 2.6 ± 1.4 DEGs/EPU; Fig. 5A, B and Supplementary Table 4). We found that 15.8% (86/543) of DEGs fell within shared EPUs and, in general, all DEGs within a single EPU showed a similar direction of misexpression (Supplementary Table 4). This finding supported the hypothesis of shared long-range, cis-acting regulation of some DEGs and highlighted the potential importance of histone modifications in this process. Chromatin immunoprecipitation followed by DNA sequencing (ChIP-seq) will be required to explore whether blastocyst transfer affects histone modifications in the placenta.

## Discussion

Evidence suggests that ART might influence pregnancy outcome in an adverse manner; yet, the mechanism is not well understood. Here we showed that blastocyst transfer after natural mating of healthy mice is sufficient to restrict placental growth at midgestation even though the associated embryos were of normal size, or even larger, on average. This phenotype corresponds with a placental transcriptome that was enriched for genes implicated in labyrinth formation, growth, vascular development, and transport function, and implies compensation by the placenta to increase its functional efficiency. Therefore, even minimal embryo manipulation by a technique used in all ART procedures has developmental implications for the placentation site and embryo. Our data suggest that changes in DNA methylation at proximal promoter regions might not disrupt gene expression in the placenta after blastocyst transfer. This is because DEGs were less likely to associate with CpG islands within a 20 kb region than genes in the genome at large and DNA methylation patterns remained unchanged in at least two DEG loci compared to non-transfer controls. The genomic clustering of some DEGs warrants further investigation of the effects of blastocyst transfer on long-range, cis-acting epigenetic mechanisms, including histone modifications and DNA methylation.

Foetal growth is contingent upon a functioning placenta since it is the interface between the maternal and foetal circulations, facilitating gas and metabolic exchange, and hormone production (Watson & Cross 2005). After blastocyst transfer, we observed placentas that were small at E10.5 suggesting delayed development and/or reduced placental growth. Since the associated embryos were of normal developmental stage and size, the placentas may have adjusted their functional performance to protect the embryo growth trajectory. To achieve this, the placenta might respond to foetal requirements by altering its structure, endocrine function, and/or nutrient transport capacity through transcriptional changes.

In the mouse, the labyrinth layer is the exchange barrier composed of a complex network of trophoblastic villi that separate the foetal capillaries from the maternal blood circulation (Watson & Cross 2005). We observed several genes associated with labyrinth formation that were upregulated after blastocyst transfer including those involved in branching morphogenesis and vascularisation as determined by genetic knockout mice (Sun *et al.* 1998, Xue *et al.* 1998, Liu & LeRoith 1999, Stumpo *et al.* 2004, Bell *et al.* 2006, Smith *et al.* 2006, Fotopoulou *et al.* 2010, Goyal *et al.* 2010, Perez-Garcia *et al.* 2018). These transcriptional changes might promote branching of additional villi and/or increased vascular density of existing villi (Wu *et al.* 2003) to improve the surface area for exchange. In support of this hypothesis, increased placental vascular density is apparent after IVF (together with embryo transfer) in bovine and ovine conceptuses (Miles *et al.* 2005, Grazul-Bilska *et al.* 2014). IVF followed by blastocyst transfer in mice might lead to increased branching morphogenesis since an initially small placenta at E12.5 develops into a large placenta by late gestation (Delle Piane *et al.* 2010, Bloise *et al.* 2012). To fully understand the effects of blastocyst transfer alone on mouse placental villus structure, a detailed morphological analysis is required. Additionally, it will be interesting to explore how specific transcriptional profiles relate to the morphological differences.

Alternatively, placentas are capable of stimulating nutrient transport to compensate for poor placental growth (Constancia *et al.* 2002). Similar to studies of whole mouse placentas at E15.5 after IVF without superovulation (Bloise *et al.* 2012), we observed many upregulated genes in transferred placentas that encode for transport proteins (e.g., *Gjb1*, gap junction protein; *Slc7a9*, L-cystine transport; *Slc27a2*, fatty acid transport; *Slc7a11*, cysteine/glutamine transport; *Apom*, lipid transport, etc.) when compared to non-transferred controls. This implies functional compensation by a small placenta in response to foetal requirements. Placental transport assays (Constancia *et al.* 2002) will be required to more fully understand the degree to which transcriptional upregulation leads to a functional increase in nutrient transport across the placenta. Furthermore, whether the transferred placenta is able to continue to compensate in later stages of gestation as the demands by the fetus increase remains to be determined.

It is possible that blastocyst transfer affects other placental regions (e.g., junctional zone or decidua). For instance, we identified four placenta prolactin cluster genes (*Prl2c2*, *Prl3d1*, *Prl7a1*, *Prl8a1*), which are solely expressed in the spongiotrophoblast cells and TGC subtypes of the mouse placenta (Simmons *et al.* 2008) and were downregulated in transferred placentas. Normally, the promoter of the *Prl3d1* gene (also known as *Pl1*) is hypomethylated in the mouse placenta compared to adult organs (Hayakawa *et al.* 2012). While DNA methylation was normal in the *Prl3d1* promoter of transferred placentas, it is possible that changes in histone modifications together with DNA methylation in a more broadly defined region surrounding the *Prl3d1* gene might be implicated. Further analysis of chromatin marks in this genomic region is required. Alternatively, reduced differentiation of progenitor cells within the ectoplacental cone of transferred conceptuses might lead to fewer parietal-TGCs (*Prl3d1*^+^ cells) and/or spongtiotrophoblast cells (*Prl2c2*^+^, *Prl7a1*^+^, or *Prl8a1*^+^ cells) at E10.5 and thus reduced expression of these genes would result. In support of this hypothesis, *Aldh3a1* and *Il33,* genes that regulate the differentiation and function of the progenitor cells within the ectoplacental cone (Nishiyama *et al.* 2015, Wang *et al.* 2017), were also downregulated in transferred placentas. To better understand the effects of blastocyst transfer on trophoblast differentiation, a histological examination and systematic assessment of trophoblast marker gene expression is required. Regardless, altered expression of prolactin cluster genes might have implications for placenta morphology, metabolism, and hormone production (Simmons *et al.* 2008, Woods *et al.* 2018).

Many researchers studying the epigenetic effects of ART have performed directed analyses of imprinted regions in placentation sites from mid-to-late gestation (reviewed by Choux *et al.* 2015) as a read-out of functional changes in genome-wide DNA methylation. Here, we performed a genome-wide approach to assess the placental transcriptome and identified only three misexpressed genes associated with distinct imprinted control regions (i.e., *Rasgrf1*, *Cobl*, *Tfpi2*) (Yoon *et al.* 2005, Monk *et al.* 2008, Shiura *et al.* 2009). Therefore, unlike more invasive ART procedures, blastocyst transfer might not alter DNA methylation to a great extent in the whole placenta at E10.5. Beyond imprinted genes and in support of this hypothesis, fewer misexpressed genes in transferred placentas were associated with intragenic and proximal promoter CpG islands than the rest of the genome implicating long-range cis-acting epigenetic mechanisms. Furthermore, we showed that at least two DEGs with known regulation by CpG methylation in the placenta (i.e., *Prl3d1* and *Rasgrf1*) (Yoon *et al.* 2005, Hayakawa *et al.* 2012) displayed normal DNA methylation patterns. A whole methylome analysis (e.g., whole genome bisulfite sequencing) will better address whether blastocyst transfer alters DNA methylation in transferred placentas to cause the widespread transcriptional changes.

Our data suggest that blastocyst transfer might disrupt other epigenetic mechanisms, such as histone modifications. The effects of ART on histone modifications are little studied and not well understood. Increased histone acetylation (e.g., H3K9ac, H3K14ac) in mouse zygotes is associated with superovulation (Huffman *et al.* 2015). Whether these changes are maintained by placenta cell lineages and influence gene expression is unclear. While we did not assess the effects of blastocyst transfer on histone modifications directly, we showed that some DEGs shared cis-acting regulatory elements in the placenta, the identification of which was largely based on the enrichment of histone modifications and RNA polymerase binding (Shen *et al.* 2012). As a result, these clustered DEGs might be sensitive to regional changes in histone modifications at key stages of development. Interestingly, four DEGs in transferred placentas (i.e., *AU022751*, *Myocd*, *Chd5*, *Pygo1*) are regulators of histone modifications (Cao *et al.* 2005, Fiedler *et al.* 2008, Potts *et al.* 2011, Zhuang *et al.* 2014, Maier *et al.* 2015, Kloet *et al.* 2016). Indeed, the dysregulation of proteins involved in chromatin remodeling has implications for widespread transcriptional dysregulation beyond the EPUs identified. Therefore, our study sets the stage for future analyses including ChIP-seq experiments, which will be required to explore the effects of blastocyst transfer on the regulation of histone modifications.

How blastocyst transfer alters placental gene expression remains unclear. The establishment of epigenetic marks is initiated at the blastocyst stage (Hanna *et al.* 2018). Therefore, it is possible that the stress of a brief culture period, embryo handling, and/or placement into a new uterine environment during this key epigenetic milestone is sufficient to alter epigenetic marks and subsequent gene expression required for cells to differentiate and function. Since all ART require blastocyst transfer, it will be important to tease apart the transcriptional and developmental effects of the transfer process from the more invasive techniques. Furthermore, the ART field will benefit from epigenome-wide analyses of placentas derived from these technologies including DNA methylation together with histone modifications with the aim of looking beyond imprinted loci. This will allow for a holistic picture of the epigenetic framework that gives rise to transcriptional and functional changes in the placenta after ART, and thus, the immediate and long-term effects on the fetus.

## Supplementary Material

Supplementary Table 1. Primer sequences (mouse)

Supplementary Table 2. DEGs with known general expression in C57Bl/6 mouse placentas at term (Yue et al. 2014)

Supplementary Table 3. DEGs with known spatial expression in mouse placentas

Supplementary Table 4. Intersection of enhancer-promoter units (EPUs) and DEGs in mouse placentas of transferred conceptuses at E10.5.

## Declaration of interest

E D W is an Associate Editor of *Reproduction*. E D W was not involved in the review or editorial process for this paper, on which she is listed as an author. The other authors have nothing to disclose.

## Funding

This work was supported by grants from the Centre for Trophoblast Research (CTR) (to E D W), Lister Institute for Preventative Medicine (to E D W), and Canadian Institutes for Health Research (to J C C). K M was funded by a Newnham College (Cambridge) studentship and A.G. Leventis scholarship. S J T was funded by a Next Generation Fellowship (CTR). G E T B was funded by a Wellcome Trust PhD studentship in Developmental Mechanisms. R S H and M P were funded by the CTR.

## Author contribution statement

E D W conceived the study. K M, R S H and E D W designed the experiments. R S H and M P performed the bioinformatic analysis. C G J, K M, M P, S J T, G E T B, and E D W performed experiments, analysed the data, and interpreted the results. E D W and J C C obtained funding. E D W and K M wrote the manuscript. All authors read and edited the manuscript.
