# Supplementary material for: Blastocyst transfer in mice alters the placental transcriptome and growth
Source: Reproduction. 2019 Nov 18;159(2):115–32. doi: 10.1530/REP-19-0293 (PMC6993209; doi:10.1530/REP-19-0293)
Supplement: Supplementary Table 1. Primer sequences (mouse) [file supplementary_table_1.pdf]

Supplementary Table 1. Primer sequences (mouse)

**RT-qPCR primers**			
**Mouse gene name**	**Forward primer (5'→3')**	**Reverse primer (5'→3')**	**Ref.**
Adamdec1	GAGGGCTTGAGAACAACCAGA	GCCCCAGAAGATGCTTGGT	-
Aldh3a1	TCCTGCTCGAGATCTTCTCTTAC	GGAGGTGCCACATGACTTTAGG	(Nishiyama *et al.* 2015)
Ang2	GAAAGGAAGCCCTTATGGACGA	ATCTGAACCCTTTAGAGGCTCG	-
Calcoco2	GGCTGCTGTTCCTGGACTT	TCAGTGAGGGTGTAATAGCACAT	-
Ccl1	GACATTCGGCGGTTGCTCTA	GTAAGCATGCTCTTGCTGTCAAC	-
Clca3a1	ACCCCGAGGCAGAGTCTTT	CACATTGGTGCCAGTGATCC	-
Cldn11	TCCCCACCTGCCGAAAAATG	ACGTAGCCTGGAAGGATGAGG	-
Ctla4	CCATGCCCGGATTCTGACTT	GGACTTCTTTTCTTTAGCATCTTGC	-
Dazl	CTAGGCAGCCACCTCACGTA	GAAGTTGTGGCAGACATGATGG	-
Eno2	CCTGGAACTAAGGGATGGGG	GGTTGTCCAGTTTCTCCTGC	-
G530011O06Rik	TTTAGGCACAGGCATCGGAA	AGCATTTCGGTGAAGCAGGA	-
Gm773	TGGAAACTTAAGCCTGCATTTGT	TGCTGTAAGTGTAGAGTGTGCT	-
Il33	AACTCCAAGATTTCCCCGGC	TTATGGTGAGGCCAGAACGG	-
Klk9	GCTGGCCTCTTCTACCTCAC	GACCCACAGGTACGGCTTTC	-
Klra7	CTCTCCAATGAGTGTAAAAGTGCAA	AGCTTTGGGGGACCAGAGTA	-
Klra8	TTCTTCTTGGAGCCTCTTAGGG	AAAATATGTCCTGTGTCTCCACC	-
Klrb1c	GAGTGTCTTAGTGCGAGTCTTAGT	TTGTGGGCACTCTAAATTAACTGAA	-
Mndal	AAGTGGAGGGGAGTGGACAA	TTGGTGACCTTGATCTTGACGA	-
Ndufa4l2	TAAAAAGACACCCTGGGCTCAT	TGGGTTGTTCTTTCTGTCCCA	-
Pianp	CACCAGGCATGCAGTAAAGG	GAGCAGGTGGTAATACGGACA	-
Prl3d1	GGAGCCTACATTGTGGTGGA	CATTCCTGCGGAGCCTGAAA	-
Ptafr	TGAGCTCCTCCTACAGGCAT	TCGGAAAGAGCGTGTATCGAA	-
Ptgdr	CCTGCCTTTAATTTATCGTGCGT	GATGAAGATCCAGGGGTCCAC	-
Rasgrf1	CAAGAGGAGTGCGGACAACC	GCGCGCGTTTACAGATACTTC	-
RNAse2a	AGACTGGGAAACATGGGTCTGG	ATGCTGGATGTCAAACCACCG	-
Tfpi2	GCTCCGTTCTTGGTCTCACT	TAGAACTTGGGGATGAGGGC	-

**Bisulfite pyrosequencing primers**			
**Location**	**Forward primer**	**Reverse Primer**	**Sequencing primer**
Prl3d1 promoter: set a	[biotin]-TTAGATTATATGGGGGATATGTAGTATG	TCCTTAAAAATTATTAACATCCTCTTTACA	ACATCCTCTTTACATTTAAC
set b	TGTGTTAAATGTAAAGAGGATGTTAATAAT	[biotin]-ACCAAACTATACCTAAACCCA	AATGTTGTTTATTAATAGATATTGA
set c	TTGGAGTAAATGTATATTGTGAGATGT	[biotin]-ACAACAACAATTACATTCCTACTAT	AAATAAGTATTTTATTAAGTAATAG
set d	ATTGTTGTTGTTGGTGTTAAGT	[biotin]-CCAAAAACAAAAACATTACTTACAAATT	TGTTATGGTGTTTATTGAAG
Rasgrf1 DMR	GGGAAGATTATTAGTTGGGGAGGTG	[biotin]-CAACAAAAACCAAAATATCAATCCTAAC	ATTAGAGTTAAATATAAAGAATGG

All primers are listed in the 5' to 3' orientation
