# Supplementary material for: Blastocyst transfer in mice alters the placental transcriptome and growth
Source: Reproduction. 2019 Nov 18;159(2):115–32. doi: 10.1530/REP-19-0293 (PMC6993209; doi:10.1530/REP-19-0293)
Supplement: Supplementary Table 2. DEGs with known general expression in C57Bl/6 mouse placentas at term (Yue et al. 2014) [file supplementary_table_2.pdf]

Supplementary Table 2. DEGs with known general expression in C57Bl/6 mouse placentas at term (Yue *et al.* 2014)

Gene name	FC	Gene function	Gene name	FC	Gene function
4930523C07Rik	2.59	Unknown, protein coding	*Jph2*	2.42	Junctional membrane complex protein
A2ml1	6.63	Peptidase inhibitor	*Kank4*	3.13	Cytoskeleton
Adamts5	2.76	Disintegrin and metalloproteinase	*Kazald1*	-2.25	Insulin-like growth factor binding
Adcy2	4.01	Adenylate cyclase	*Kcne3*	3.91	Potassium channel activity
Aff2	2.51	Transcriptional activator	*Klb*	3.97	FGF signaling pathway
Adgre1	2.03	G protein-coupled hormone receptor	*Klrd1*	-2.71	Killer cell lectin-like receptor; cell adhesion
Aldh1l2	-2.21	One-carbon metabolism (mitochondria)	*Krt23*	2.19	Intermediate filament
Apbb1ip	-2.13	Ras signaling pathway	*Kynu*	4.64	NAD cofactor biosynthesis
Apobec1	2.57	Cytodine deaminase	*Ltb4r1*	-2.71	G protein-coupled receptor activity
Arhgef26	2.37	Rho-guanine nucleotide exchange factor	*Lst1*	2.09	Membrane protein
Arsk	3.00	Sulfatase, hormone biosynthesis	*Mid1*	-2.27	Cytoskeleton
B3gat2	-2.55	Carbohydrate metabolism	*Mndal*	-2.96	RNA polymerase
Btg3	-2.86	Transcription factor binding protein	*Ms4a4b*	-3.71	Membrane protein
C1qtnf1	2.21	MAPK pathway	*Ms4a6c*	2.09	Membrane protein
Ccdc3	2.29	TNFα regulation	*Myocd*	3.61	Transcription factor
Cd53	-2.17	Integrin binding, involved in cell growth	*Naip6*	-2.17	Apoptosis inhibitory protein
Cmc4	-2.00	Mitochondrial protein import	*Ntrk1*	-2.32	Kinase in MAPK pathway
Chmp4c	3.89	Chromatin modifying protein, mitosis	*Osm*	-2.04	Secreted cytokine; growth regulator
Clca3a1	-3.90	Calcium-activated chloride channel	*Otoa*	-2.63	Predicted adhesion protein
Cldn1	3.30	Tight junction	*Pcdhga1*	2.52	Protocadherin
Cfb	4.40	Cell proliferation	*Pcdhgb7*	2.29	Protocadherin
Cobl	2.61	Actin nucleator	*Pianp*	50.8	Cell adhesion
Colgalt2	5.27	Extracellular matrix; PPARγ pathway	*Pllp*	2.37	Ion transport
Crabp2	2.32	Retinoic acid signaling pathway	*Ptafr*	4.85	G-protein coupled receptor activity
Csdc2	4.62	RNA-binding factor; histone synthesis	*Ptgs2*	-2.25	Prostaglandin synthesis
Ctla4	-5.52	Cytotoxic T-lymphocyte-associated protein	*Rai2*	2.00	Retinoic acid signaling pathway
Dpp4	2.32	Adenosine deaminase	*Rasgrf1*	-2.70	Ras protein signal transduction pathway
Efcab7	2.36	Hedgehog signalling	*Rgs1*	-4.34	Regulator of G protein signaling
Egln3	-2.45	HIF-1 signaling pathway	*Rnf150*	7.53	Ubiquitin protein ligase activity
Eno1b	4.73	Glycolysis	*Robo2*	3.41	Cell migration
Enpep	2.20	Aminopeptidase	*Schip1*	2.13	Estrogen metabolism
Fam20a	2.13	Unknown	*Slc7a11*	2.57	Cysteine and glutamate transport
Fam189a2	3.20	Unknown	*Slc9a2*	2.87	Na^+/H^+ transporter
Fam196a	3.89	Unknown	*Slc27a2*	2.60	Fatty acid transport
Far2	-2.03	fatty-acyl-CoA reductase	*Slc28a2*	-2.03	Na-coupled purine nucleoside

		activity				transporter
Fbn1	2.38	Fibrillin		*Slit3*	2.32	Slit/Robo pathway
Galnt2	2.95	Glycosyltransferase		*Sorbs1*	2.28	Insulin signaling
Gpr19	2.46	G protein coupled receptor		*Spon1*	2.39	Cell adhesion
Gpr141	-2.27	Rhodopsin G protein-coupled receptor		*Stom*	2.12	Membrane protein
Gpr183	-3.60	G protein-coupled receptor		*Sytl3*	-2.53	Exocytosis
Grap2	2.32	GRB2-related adaptor protein		*Tfpi*	-2.57	Tissue factor pathway inhibitor
Gucy2c	-2.34	cGMP biosynthesis		*Thbs1*	2.15	Thrombospondin
Gxylt2	3.03	EGF pathway		*Thbs2*	2.69	Thrombospondin
Hipk2	2.19	Negative regulator of BMP pathway		*Trim5*	-2.01	E3 ubiquitin-ligase
Hkdc1	2.57	Glucose metabolism		*Trim55*	-2.37	Cytoskeleton
Hnf4a	2.66	Transcription factor		*Trpm2*	-10.1	Cation channel
Hspb7	16.7	Chaperone		*Tspan8*	3.46	Integrin binding
Ifi205	-2.15	Transcription factor		*Unc93a*	2.95	Unknown
Il15ra	-2.63	Interleukin		*Wdfy1*	2.67	Phosphatidylinositol 3-phosphate binding protein
Il33	4.04	Interleukin		*Wscd1*	3.07	Sulfotransferase activity
Itgb7	2.34	Integrin; cell-ECM adhesion		*Zfand4*	-2.19	Zinc finger protein

FC, fold change
