# Supplementary material for: Blastocyst transfer in mice alters the placental transcriptome and growth
Source: Reproduction. 2019 Nov 18;159(2):115–32. doi: 10.1530/REP-19-0293 (PMC6993209; doi:10.1530/REP-19-0293)
Supplement: Supplementary Table 3. DEGs with known spatial expression in mouse placentas [file supplementary_table_3.pdf]

Supplementary Table 3. DEGs with known spatial expression in mouse placentas

Gene name	FC	Gene function	Reported placental expression in mouse (stage of development)	Ref.
Expression in multiple cell types				
Cp	2.63	Iron homeostasis	Lab TB, SpA-TGC, FVE, PE, Dec (E14.5)	(Han *et al.* 2018)
Guca2b	4.67	cGMP biosynthesis	Lab TB (E14.5); Dec (E7.5)	(McConaha *et al.* 2011, Han *et al.* 2018)
Lcn2	3.34	Lipocalin 2	Lab TB (E14.5); Dec (E7.5)	(McConaha *et al.* 2011, Han *et al.* 2018)
Ltf	2.28	Lactotransferrin	Lab TB (E14.5); Dec (E7.5)	(McConaha *et al.* 2011, Han *et al.* 2018)
Tnfrsf11b	2.17	Tumor necrosis factor-receptor	Lab TB, Dec (E14.5)	(Han *et al.* 2018)
Gldn	-3.77	Extracellular matrix	Lab TB, PE (E14.5)	(Han *et al.* 2018)
Trpv6	3.68	Calcium transport	Lab TB (S-TGCs), EPC (GlyT) (E17.5)	(Yang *et al.* 2015)
Ang2	799.3	Ribonuclease	Lab TB (S-TGCs), FVE (E17.5)	(Geva *et al.* 2005)
Aldh3a1	-9.29	Aldehyde dehydrogenase	TS cells (in vitro), EPC (SpT) (E11.5-E17.5), Dec (E7.5)	(McConaha *et al.* 2011, Nishiyama *et al.* 2015)
Emp1	2.20	Cell adhesion	FVE, PE/EC (E14.5)	(Han *et al.* 2018)
Fabp4	2.15	Fatty acid binding protein	FVE, Dec (E12.5-E18.5)	(Makkar *et al.* 2014, Han *et al.* 2018)
Rnase4	4.43	Ribonuclease	PE/EC, Dec (E14.5)	(Han *et al.* 2018)
Zfp36l1	2.06	Zinc finger protein	Allantois, YS (E8.0-E9.5); Dec, (E14.5)	(Stumpo *et al.* 2004, Bell *et al.* 2006, Han *et al.* 2018)
Tmem26	3.20	Transmembrane protein	HPSC, Stromal cell (E14.5)	(Han *et al.* 2018)
Trophoblast progenitor cell only expression				
Gm1821	-2.31	Unknown; protein coding	Trophoblast progenitor (E14.5)	(Han *et al.* 2018)
Labyrinth trophoblast cells only expression				
Pigr	5.93	EGF pathway	Lab TB (E14.5)	(Han *et al.* 2018)
C3	4.60	Complement system; ERK1/2 cascade	Lab TB (E14.5)	(Han *et al.* 2018)
Sprr2f	4.09	Epidermis development	Lab TB (E14.5)	(Han *et al.* 2018)
Sftpd	3.91	Carbohydrate binding, superfactant homeostasis	Lab TB (E14.5)	(Han *et al.* 2018)
Fbln2	3.84	Extracellular matrix	Lab TB (E14.5)	(Han *et al.* 2018)
Prap1	3.35	Proline rich acidic protein	Lab TB (E14.5)	(Han *et al.* 2018)
Fermt1	3.12	Cell adhesion	Lab TB (E14.5)	(Han *et al.* 2018)
Robo1	2.90	Slit receptor, cell migration	S-TGCs (E13.5, E15.5)	(Li *et al.* 2015)
Srd5a1	-2.52	Steroid 5 α-reductase	Lab TB (E14.5)	(Han *et al.* 2018)
Napsa	-2.36	Aspartic protease	Lab TB (E14.5)	(Han *et al.* 2018)
Slc39a8	2.31	Zinc ion transporter	Lab TB (E14.5)	(Han *et al.* 2018)
EPC trophoblast lineage only expression				
Inhba	4.42	TGFβ signaling pathway	EPC, P-TGCs (E7.5)	(Albano *et al.* 1994)
Prl8a1	-2.89	Prolactin cluster	EPC, SpT, P-TGCs (E12.5-E18.5)	(Simmons *et al.* 2008, Han *et al.* 2018)
Prl7a1	-2.85	Prolactin cluster	EPC, SpT, P-TGCs (E8.5-E15.5)	(Simmons *et al.* 2008, Han *et al.* 2018)
Prl2c2	-2.27	Prolactin cluster (proliferin)	EPC, SpT, P-TGCs (E8.5-E18.5)	(Simmons *et al.* 2008)

Prl3d1	-2.02	Prolactin cluster	P-TGCs (E8.5-E10.5)	(Simmons *et al.* 2008)
Fetal vascular endothelial (FVE) cell only expression				
Enpp2	2.48	Ectonucleotide pyrophosphatase/ phosphodiesterase	FVE (E14.5)	(Han *et al.* 2018)
Parietal endodermal (PE) cells and endodermal cells (EC) only expression				
H2-Q10	13.01	Cell adhesion, antigen processing	EC (E14.5)	(Han *et al.* 2018)
Edn2	7.15	Angiogenesis	PE (E14.5)	(Han *et al.* 2018)
Itih2	4.03	Serine protease; ECM stabilization	EC (E14.5)	(Han *et al.* 2018)
Ambp	4.01	Peptidase inhibitor	EC (E14.5)	(Han *et al.* 2018)
Apob	3.74	Apolipoprotein	EC (E14.5)	(Han *et al.* 2018)
Myl7	-3.71	Focal adhesions	PE (E14.5)	(Han *et al.* 2018)
Lrp2	3.43	Multi-ligand endocytic receptor	EC (E14.5)	(Han *et al.* 2018)
F2	3.37	Vascular integrity	EC (E14.5)	(Han *et al.* 2018)
Fbp2	3.25	Fructose bisphosphotase	PE (E14.5)	(Han *et al.* 2018)
Serpind1	3.07	Inhibitor of proteases	EC (E14.5)	(Han *et al.* 2018)
Cubn	2.89	Receptor mediating endocytosis	EC (E14.5)	(Han *et al.* 2018)
Gjb1	2.84	Gap junction protein	EC (E14.5)	(Han *et al.* 2018)
Slc7a9	2.72	Cysteine transport	EC (E14.5)	(Han *et al.* 2018)
Kng2	2.32	Kininogenin	EC (E14.5)	(Han *et al.* 2018)
Mttp	2.30	Lipoprotein assembly	EC (E14.5)	(Han *et al.* 2018)
Cldn2	2.29	Tight junction protein	EC (E14.5)	(Han *et al.* 2018)
Ang	2.21	Angiogenesis	PE, EC (E14.5)	(Han *et al.* 2018)
Aifm3	2.14	Oxidoreductase, apoptotic process	PE (E14.5)	(Han *et al.* 2018)
Apom	2.09	Apoplipoprotein	EC (E14.5)	(Han *et al.* 2018)
Sod3	2.08	Superoxide dismutase	EC (E14.5)	(Han *et al.* 2018)
Amn	2.04	Cobalamin transport	EC (E14.5)	(Han *et al.* 2018)
Ctsf	2.02	Cathepsin	PE (E14.5)	(Han *et al.* 2018)
Stromal cell only expression				
Sfrp2	2.84	WNT signaling pathway	Stromal cell (E14.5)	(Han *et al.* 2018)
Igf1	2.23	Insulin-like growth factor signaling	Stromal cell (E14.5)	(Han *et al.* 2018)
Fxyd1	-2.02	Ion channel protein	Stromal cell (E14.5)	(Han *et al.* 2018)
Hematopoietic stem cell (HPSC) only expression				
Gm14165	3.30	Unknown; pseudogene	HPSC (E14.5)	(Han *et al.* 2018)
Klk8	-3.25	Serine protease	HPSC (E14.5)	(Han *et al.* 2018)
Ifitm1	-2.83	Interferon induced transmembrane protein	HPSC (E14.5)	(Han *et al.* 2018)
Cd69	-2.13	Calcium and carbohydrate binding	HPSC (E14.5)	(Han *et al.* 2018)
Rps23	-2.06	Ribosomal protein	HPSC (E14.5)	(Han *et al.* 2018)
Mir703	-2.05	MicroRNA	HPSC (E14.5)	(Han *et al.* 2018)
Fetal erythroid cell only expression				
Slc4a1	2.03	Chloride/bicarbonate exchanger	Fetal erythroid cell (E14.5)	(Han *et al.* 2018)
Decidua (Dec) cell only expression				
Pdgfrl	14.46	PDGF receptor-like protein	Dec (E7.5)	(Ashley *et al.* 2010)
Fcgbp	9.67	Fc fragment of IgG binding protein	Dec (E7.5)	(McConaha *et al.* 2011)
Cldn11	5.87	Tight junction	Dec (E14.5)	(Han *et al.* 2018)

Sfrp5	4.15	WNT signaling pathway	Dec (E14.5)	(Han *et al.* 2018)
Cdo1	4.08	SHH co-receptor	Dec (E10.5, E14.5)	(Rakoczy *et al.* 2015, Han *et al.* 2018)
Erv3	3.27	Endogenous retrovial sequence	Dec (E7.5)	(McConaha *et al.* 2011)
Tfpi2	3.12	Serine protease involved in tissue remodelling	Dec (E14.5)	(Han *et al.* 2018)
Ear2	3.04	Ribonuclease	Dec (E7.5)	(McConaha *et al.* 2011)
Slpi	2.47	Serine protease inhibitor	Dec (E14.5)	(Han *et al.* 2018)
Aqp1	2.32	Aquaporin	Dec (E14.5)	(Han *et al.* 2018)
Uterine natural killer cell (uNK) expression				
Klrb1b	-3.27	Killer cell lectin-like receptor	uNK cells (14.5)	(Han *et al.* 2018)
Tff1	-2.75	Secreted protein	uNK cells (E14.5)	(Han *et al.* 2018)
Gzmn	-2.23	Endopeptidase	uNK cells (E14.5)	(Han *et al.* 2018)
Gzmb	-2.18	Hydrolyase	uNK cell (E14.5)	(Han *et al.* 2018)
Trbc1	2.00	T cell receptor	uNK cell (E14.5)	(Han *et al.* 2018)

Dec, decidua; E, embryonic day; EC, endodermal cell; ECM, extracellular matrix; EPC, ectoplacental cone; FC, fold change; FVE, fetal vascular endothelium; GlyT, glycogen trophoblast cell; HSPC, hematopoietic stem cell; Lab troph, undefined labyrinth trophoblast cell; PE, parietal endoderm; P-TGCs, parietal TGC; SMA cell, smooth muscle actin cell; SpT, spongiotrophoblast cells; SpA-TGC, spiral artery TGC; S-TGC, sinusoidal TGC; TGC, trophoblast giant cell; TS cell, trophoblast stem cell; YS, yolk sac.

References

Albano RM, Arkell R, Beddington RS & Smith JC 1994 Expression of inhibin subunits and follistatin during postimplantation mouse development: decidual expression of activin and expression of follistatin in primitive streak, somites and hindbrain. *Development* **120** 803-813.

Ashley RL, Henkes LE, Bouma GJ, Pru JK & Hansen TR 2010 Deletion of the Isg15 gene results in up-regulation of decidual cell survival genes and down-regulation of adhesion genes: implication for regulation by IL-1beta. *Endocrinology* **151** 4527-4536.

Bell SE, Sanchez MJ, Spasic-Boskovic O, Santalucia T, Gambardella L, Burton GJ, Murphy JJ, Norton JD, Clark AR & Turner M 2006 The RNA binding protein Zfp36l1 is required for normal vascularisation and post-transcriptionally regulates VEGF expression. *Dev Dyn* **235** 3144-3155.

Geva E, Ginzinger DG, Moore DH, 2nd, Ursell PC & Jaffe RB 2005 In utero angiopoietin-2 gene delivery remodels placental blood vessel phenotype: a murine model for studying placental angiogenesis. *Mol Hum Reprod* **11** 253-260.

Han X, Wang R, Zhou Y, Fei L, Sun H, Lai S, Saadatpour A, Zhou Z, Chen H, Ye F*, et al.* 2018 Mapping the Mouse Cell Atlas by Microwell-Seq. *Cell* **172** 1091-1107 e1017.

Li P, Peng H, Lu WH, Shuai HL, Zha QB, Yeung CK, Li H, Wang LJ, Ho Lee KK, Zhu WJ*, et al.* 2015 Role of Slit2/Robo1 in trophoblast invasion and vascular remodeling during ectopic tubal pregnancy. *Placenta* **36** 1087-1094.

Makkar A, Mishima T, Chang G, Scifres C & Sadovsky Y 2014 Fatty acid binding protein-4 is expressed in the mouse placental labyrinth, yet is dispensable for placental triglyceride accumulation and fetal growth. *Placenta* **35** 802-807.

McConaha ME, Eckstrum K, An J, Steinle JJ & Bany BM 2011 Microarray assessment of the influence of the conceptus on gene expression in the mouse uterus during decidualization. *Reproduction* **141** 511-527.

Nishiyama M, Nita A, Yumimoto K & Nakayama KI 2015 FBXL12-Mediated Degradation of ALDH3 is Essential for Trophoblast Differentiation During Placental Development. *Stem Cells* **33** 3327-3340.

Rakoczy J, Lee S, Weerasekera SJ, Simmons DG & Dawson PA 2015 Placental and fetal cysteine dioxygenase gene expression in mouse gestation. *Placenta* **36** 956-959.

Simmons DG, Rawn S, Davies A, Hughes M & Cross JC 2008 Spatial and temporal expression of the 23 murine Prolactin/Placental Lactogen-related genes is not associated with their position in the locus. *BMC Genomics* **9** 352.

Stumpo DJ, Byrd NA, Phillips RS, Ghosh S, Maronpot RR, Castranio T, Meyers EN, Mishina Y & Blackshear PJ 2004 Chorioallantoic fusion defects and embryonic lethality resulting from disruption of Zfp36L1, a gene encoding a CCCH tandem zinc finger protein of the Tristetraprolin family. *Mol Cell Biol* **24** 6445-6455.

Yang H, Ahn C & Jeung EB 2015 Differential expression of calcium transport genes caused by COMT inhibition in the duodenum, kidney and placenta of pregnant mice. *Mol Cell Endocrinol* **401** 45-55.
