# Supplementary material for: Blastocyst transfer in mice alters the placental transcriptome and growth
Source: Reproduction. 2019 Nov 18;159(2):115–32. doi: 10.1530/REP-19-0293 (PMC6993209; doi:10.1530/REP-19-0293)
Supplement: Supplementary Table 4. Intersection of enhancer-promoter units (EPUs) and DEGs in mouse placentas of transferred conceptuses at E10.5. [file supplementary_table_4.pdf]

Supplementary Table 4. Intersection of enhancer-promoter units (EPUs) and DEGs in mouse placentas of transferred conceptuses at E10.5.

EPU	chr	Gene name	ensembl_gene_id	FC	padj
EPU_1	chr1	*Gm15850*	ENSMUSG00000086264	2.06	0.044692317
		Gm26781	ENSMUSG00000097433	2.36	0.001894297
EPU_2	chr1	*4930523C07Rik*	ENSMUSG00000090394	2.59	6.00E-19
		Tnn	ENSMUSG00000026725	4.15	1.40E-07
EPU_3	chr1	*Itln1*	ENSMUSG00000038209	246.27	9.25E-12
		Ly9	ENSMUSG00000004707	2.04	0.004582751
EPU_4	chr1	*Ifi213*	ENSMUSG00000073491	2.70	6.65E-06
		Ifi208	ENSMUSG00000066677	3.95	3.73E-05
		Mndal	ENSMUSG00000090272	2.96	3.43E-33
		Ifi202b	ENSMUSG00000026535	40.50	4.86E-48
		Ifi205	ENSMUSG00000054203	2.15	0.009521903
EPU_5	chr2	*AI847159*	ENSMUSG00000084826	3.05	1.94E-11
		Gm14029	ENSMUSG00000086652	2.15	1.11E-16
EPU_6	chr3	*Sprr2f*	ENSMUSG00000050635	4.09	2.24E-73
		Sprr2g	ENSMUSG00000046203	5.19	8.43E-08
EPU_7	chr3	*Selenbp2*	ENSMUSG00000068877	11.23	3.30E-08
		Gm15264	ENSMUSG00000081355	11.02	6.06E-22
EPU_8	chr3	*Fam46c*	ENSMUSG00000044468	3.04	2.95E-246
		Gm12474	ENSMUSG00000053957	2.44	1.33E-05
EPU_9	chr4	*Edn2*	ENSMUSG00000028635	7.15	4.73E-06
		Foxo6	ENSMUSG00000052135	5.33	0.040654175
EPU_10	chr4	*Clcnkb*	ENSMUSG00000006216	75.48	0.019122743
		Hspb7	ENSMUSG00000006221	16.67	4.32E-48
EPU_11	chr4	*Chd5*	ENSMUSG00000005045	2.63	7.50E-42
		Kcnab2	ENSMUSG00000028931	2.02	1.06E-08
EPU_12	chr6	*Trbc1*	ENSMUSG00000076490	2.00	8.37E-49
		Trpv6	ENSMUSG00000029868	3.68	3.16E-13
EPU_13	chr6	*Aicda*	ENSMUSG00000040627	6.72	1.90E-08
		Apobec1	ENSMUSG00000040613	2.58	2.92E-10
EPU_14	chr6	*Klrb1c*	ENSMUSG00000030325	9.71	5.19E-16
		Klrb1b	ENSMUSG00000079298	3.27	6.46E-17
EPU_14	chr6	*Clec2i*	ENSMUSG00000030365	3.02	1.62E-48
		Klre1	ENSMUSG00000050241	2.63	0.017626571
EPU_15	chr6	*Klrd1*	ENSMUSG00000030165	2.71	0.001680801
		Klra4	ENSMUSG00000079852	8.83	2.84E-05
		Klra8	ENSMUSG00000089727	6.82	1.60E-46
		Klra14-ps	ENSMUSG00000072721	8.07	0.001104723
		Klra7	ENSMUSG00000067599	5.60	0.023579355
		Klra13-ps	ENSMUSG00000030178	2.87	2.64E-05
		Styk1	ENSMUSG00000032899	2.19	0.000660299
EPU_16	chr7	*Klk9*	ENSMUSG00000047884	4.07	0.022546162
		Klk8	ENSMUSG00000064023	3.25	0.047080871
EPU_17	chr7	*Trim5*	ENSMUSG00000060441	2.01	0.000295991
		Trim12a	ENSMUSG00000066258	2.14	6.65E-05
		Trim30d	ENSMUSG00000057596	2.04	7.85E-07

EPU_18	chr8	*Bco1*	ENSMUSG00000031845	2.55	9.37E-07
		Gm20694	ENSMUSG00000093446	3.07	1.54E-21
EPU_19	chr9	*Gm7257*	ENSMUSG00000023093	7.67	0.01393948
		Gm9513	ENSMUSG00000090710	7.90	5.04E-08
EPU_20	chr9	*Jhy*	ENSMUSG00000032023	3.04	0.004658411
		Crtam	ENSMUSG00000032021	2.83	1.88E-06
EPU_21	chr13	*Prl8a1*	ENSMUSG00000019756	2.90	4.23E-11
		Prl7a1	ENSMUSG00000006488	2.85	0.008311304
EPU_22	chr14	*Rnase4*	ENSMUSG00000021876	4.43	1.62E-05
		Ang	ENSMUSG00000072115	2.21	7.06E-09
EPU_23	chr14	*Gzmn*	ENSMUSG00000015443	2.23	0.00522498
		Gzmb	ENSMUSG00000015437	2.18	0.006425581
EPU_24	chr14	*Adamdec1*	ENSMUSG00000022057	1194.78	4.01E-08
		Adam28	ENSMUSG00000014725	57.59	3.56E-09
EPU_25	chr17	*Tff1*	ENSMUSG00000024032	2.75	1.50E-24
		Ubash3a	ENSMUSG00000042345	2.01	0.022452667
EPU_26	chr17	*Lst1*	ENSMUSG00000073412	2.09	4.01E-05
		H2-Q1	ENSMUSG00000079507	16.56	2.96E-08
		H2-Q2	ENSMUSG00000091705	29.40	0.000132378
		H2-Q5	ENSMUSG00000055413	2.06	4.96E-13
		H2-Q10	ENSMUSG00000067235	13.01	0.001118777
		Cdsn	ENSMUSG00000039518	3.06	1.38E-25
EPU_27	chr17	*H2-T24*	ENSMUSG00000053835	2.69	0.014453217
		Gm11127	ENSMUSG00000079492	164.59	4.34E-10
		Gm8810	ENSMUSG00000091373	537.65	0.000572567
		Gm10499	ENSMUSG00000073403	573.81	2.17E-14
		Gm8909	ENSMUSG00000073402	70.98	0.000951945
EPU_28	chr18	*Pcdhga1*	ENSMUSG00000103144	2.52	1.05E-05
		Pcdhgb7	ENSMUSG00000104063	2.29	2.60E-06
EPU_29	chr19	*Ctsf*	ENSMUSG00000083282	2.02	6.71E-05
		Actn3	ENSMUSG00000006457	8.40	0.028619096
EPU_30	chr19	*Ms4a4b*	ENSMUSG00000056290	3.71	0.025157384
		Ms4a6c	ENSMUSG00000079419	2.09	0.018793207
EPU_31	chr19	*Sfrp5*	ENSMUSG00000018822	4.15	0.002602491
		Golga7b	ENSMUSG00000042532	2.41	0.035772232
EPU_32	chrX	*Gm5127*	ENSMUSG00000073010	4.69	0.026925397
		P2ry10	ENSMUSG00000050921	3.55	1.09E-05
		A630033H20Rik	ENSMUSG00000054293	2.11	0.046901925
EPU_33	chrX	*Mid1*	ENSMUSG00000035299	2.27	1.41E-05
		G530011O06Rik	ENSMUSG00000072844	14.49	1.93E-10
		Gm15726	ENSMUSG00000087263	4.86	0.002939615

See also Figure 5. Chr, chromosome; EPU, enhancer-promoter unit; FC, fold change.
